# HIV-1 Suppressive Sequences Are Modulated by Rev Transport of Unspliced RNA and Are Required for Efficient HIV-1 Production

**DOI:** 10.1371/journal.pone.0051393

**Published:** 2012-12-10

**Authors:** Kousei Noguchi, Keisuke Ishibashi, Kaori Miyokawa, Manami Hokari, Tomoyuki Kanno, Tomoya Hirano, Norio Yamamoto, Hiroshi Takaku

**Affiliations:** 1 Department of Life and Environmental Science, Chiba Institute of Technology, Tsudanuma, Narashino-shi, Chiba, Japan; 2 High Technology Research Center, Chiba Institute of Technology, Tsudanuma, Narashino-shi, Chiba, Japan; 3 Influenza Virus Research Center, National Institute of Infectious Diseases, Musashimurayama-shi, Tokyo, Japan; International Centre for Genetic Engineering and Biotechnology, Italy

## Abstract

The unspliced human immunodeficiency virus type 1 (HIV-1) RNAs are translated as Gag and Gag-Pol polyproteins or packaged as genomes into viral particles. Efficient translation is necessary before the transition to produce infective virions. The viral protein Rev exports all intron-containing viral RNAs; however, it also appears to enhance translation. Cellular microRNAs target cellular and viral mRNAs to silence their translation and enrich them at discrete cytoplasmic loci that overlap with the putative interim site of Gag and the genome. Here, we analyzed how Rev-mediated transport and the splicing status of the mRNA influenced the silencing status imposed by microRNA. Through identification and mutational analysis of the silencing sites in the HIV-1 genome, we elucidated the effect of silencing on virus production. *Renilla* luciferase mRNA, which contains a let-7 targeting site in its 3′ untranslated region, was mediated when it was transported by Rev and not spliced, but it was either not mediated when it was spliced even in a partial way or it was Rev-independent. The silencing sites in the *pol* and *env*-*nef* regions of the HIV-1 genome, which were repressed in T cells and other cell lines, were Drosha-dependent and could also be modulated by Rev in an unspliced state. Mutant viruses that contained genomic mutations that reflect alterations to show more derepressive effects in the 3′ untranslated region of the *Renilla* luciferase gene replicated more slowly than wild-type virus. These findings yield insights into the HIV-1 silencing sites that might allow the genome to avoid translational machinery and that might be utilized in coordinating virus production during initial virus replication. However, the function of Rev to modulate the silencing sites of unspliced RNAs would be advantageous for the efficient translation that is required to support protein production prior to viral packaging and particle production.

## Introduction

Human immunodeficiency virus type 1 (HIV-1) is unique in that it produces two RNA types: Rev-independent and Rev-dependent. Completely spliced RNA is exported to the cytoplasm as cellular mRNA. In contrast, incompletely spliced, unspliced and genomic RNAs retain a structured RNA region known as the Rev response element (RRE), which is used by Rev to transport these RNA species into the cytoplasm in a Crm1-dependent manner [1]. An increased RNA transport rate also enhances translational efficiency. However, Rev also appears to enhance translation [2], [3] and packaging efficiency [4]–[6] in the cytoplasm.

The unspliced HIV-1 RNAs are translated as Gag and Gag-Pol polyproteins or are packaged as genomes into viral particles. Both Gag and the viral genome are transported in late endosomal structures called multivesicular bodies (MVBs) to the plasma membrane for virus egress, a process that requires the participation of the Endosomal Sorting Complex Required for Transport (ESCRT) proteins [7]–[11]. Packaging requires Gag and its specific recognition of the HIV-1 genome. The packaging site has not been precisely determined; however, the optimization of viral output requires a translation and packaging equilibrium [12], [13]. The HIV-1 genome must be sequestered from the cellular translational machinery to be packaged into viral particles.

MicroRNAs (miRNAs) are approximately 21-nt small RNAs produced via cleavage by the RNase III enzymes Drosha and Dicer. Next, miRNAs guide the RNA-induced silencing protein complex (RISC) to partially complementary mRNAs, leading to the degradation and/or translational silencing of the targeted mRNA [14]. There appears to be a greater relative concentration of RISC-incorporated miRNA and targeted RNA in MVBs [15]–[17]. Because HIV-1 uses MVBs and ESCRT components for replication, it is reasonable to suggest that HIV-1 might usurp host RNA silencing mechanisms [17]. A mechanism may exist that controls the equilibrium between packaging and translation efficiency, but a long unspliced HIV-1 RNA would still have to be effective in producing Gag and Gag-Pol polyproteins. In this context, it has yet to be elucidated whether Rev-dependent unspliced RNAs influence miRNA-mediated silencing differently than completely spliced and cellular mRNAs.

The HIV-1 genome is targeted by several miRNAs, and cellular miRNA appears to be preferentially upregulated upon HIV-1 infection [18], [19]. In fact, HIV-1 appears to utilize miRNA silencing to maintain a latent infection in resting CD4^+^ T cells, suggesting the miRNA profile of resting CD4^+^ T cells favors HIV-1 latency [18]. Therefore, while miRNAs might promote HIV-1 survival *in vivo*, miRNAs appear to have adverse effects on viral replication, at least when the candidate miRNA is overexpressed in cultured cell lines [20]. However, different results might be obtained if the target sequence within the virus is mutated [21]. To improve our understanding of the relationship between HIV-1 and cellular miRNAs, we identified potential regions of the HIV-1 genome that might be susceptible to silencing via targeting after insertion into the 3′ untranslated region (3′ UTR) of the *Renilla* luciferase (*Rluc*). We then assessed whether HIV-1 replication was affected by silent mutations in the silencing sites.

In this study, we addressed the role of splicing and transport mechanisms in the regulation of miRNA-mediated silencing of Rev-dependent and Rev-independent RNAs. In addition, we identified the suppressive sites within the HIV-1 genome and addressed whether these sites revealed any HIV-1 genome effects during virus replication and whether the effect was modulated by Rev. Our results suggest that suppressive sequences may promote initial virus replication and may be coordinated by Rev-inhibited RNA silencing.

## Results

### The Effects of Let-7-mediated Interference on mRNAs are Regulated by Splicing and Rev-mediated mRNA Export

**Figure 1:**
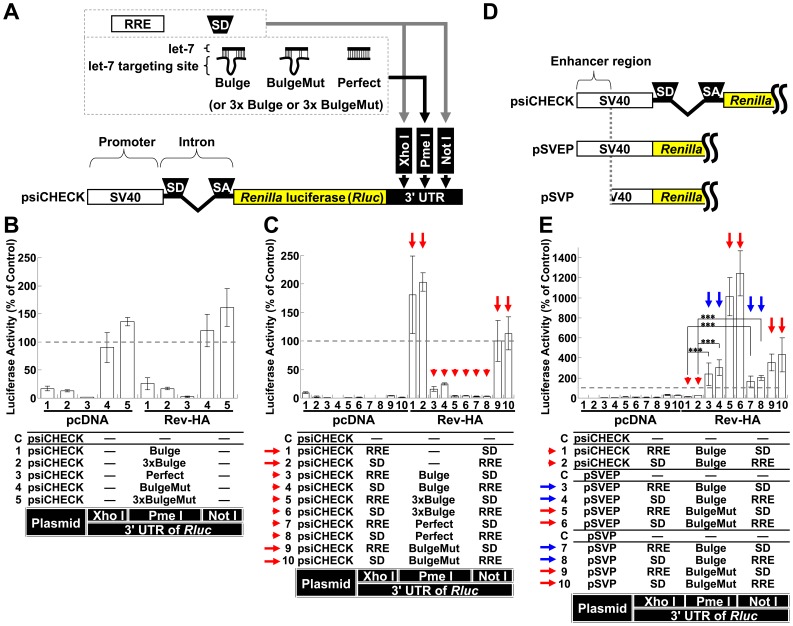
The role of splicing and Rev-dependent export on miRNA silencing. (A) A schematic of the SV40 promoter, intron and 3′ UTR from which the *Renilla* luciferase (*Rluc*) was transcribed in the psiCHECK-2 vector (psiCHECK). “SD” and “SA” indicate the splice donor and splice acceptor sites, respectively. The 3′ UTR has three restriction sites (XhoΙ, PmeΙ and NotΙ). Each let-7 targeting sequence was inserted into the PmeΙ site, and the “SD” or Rev response element (RRE) was inserted into the XhoΙ or NotΙ sites in various combinations. (B) The silencing of the RNA and mutated sequences cloned into the PmeΙ restriction site in the *Rluc* 3′ UTR in the presence of Rev-HA was assessed. The *Rluc* activity was normalized to the firefly luciferase activity, and an empty vector C was used as a control. (C) The miRNA-mediated silencing of the spliced Rev-HA-exported RNAs was assessed. “RRE” and “SD” were inserted into the restriction sites (XhoΙ or NotΙ) in the *Rluc* 3′ UTR. The red arrow points to the vectors that presented altered *Rluc* activity in the presence of Rev-HA. The red arrowhead points to the Bulge-, 3×Bulge- or Perfect-containing constructs that carry a correctly oriented RRE and were silenced in the presence of Rev-HA. (D) A schematic of the SV40 promoter and intron region and the truncated promoters without an intron. (E) The effects of splicing and the presence of enhancers on miRNA-mediated silencing were analyzed using plasmids containing the SV40 promoter or its truncated versions. The blue arrow points to the Bulge-containing constructs without an intron that carry a correctly oriented RRE and were not silenced in the presence of Rev-HA. For each promoter tested, the empty vector C was used as a control. The *Renilla*/firefly luciferase value was assessed, and six independent experiments were performed and expressed as the mean ± S.D. as a percentage of the control. ***P<0.001.

To address the effects of miRNA-mediated interference on the expression from spliced and unspliced RNAs in the presence and absence of Rev, we initially analyzed the spliced mRNAs, including *tat* and *rev*. To quantitatively determine the extent of repression, the expression of *Renilla* luciferase (*Rluc*) from the psiCHECK-2 vector (psiCHECK) was used as a spliced mRNA model, through the introduction of a chimeric intron that was originally inserted to augment expression (**Fig. 1A**). The let-7 binding sequence was selected as the target because it has been successfully used in HeLa cells with abundant expression [22]. A single let-7 binding sequence (Bulge), three consecutive binding sequences (3×Bulge), a perfectly complementary sequence (Perfect; used to trigger siRNA interference), a mutated sequence (BulgeMut) and three consecutive mutated sequences (3×BulgeMut) were individually inserted into the PmeΙ restriction site of the *Rluc* 3′ UTR (**Fig. 1A and B**). As expected, a modest repressive effect on luciferase activity was observed in cells transfected with the Bulge and 3×Bulge constructs (Vectors 1 and 2), while repression was highly efficient in the cells transfected with the Perfect construct (Vector 3). The coexpression of Rev-HA (**Fig. S1A**) did not significantly influence the suppressive effects of the let-7 binding site (**Fig. 1B** **,** Vectors 1–3 in the presence of Rev-HA).

To examine the effects of miRNA-mediated suppression on partially spliced mRNAs, such as *env* and *vpr*, a Rev response element (RRE) and a splice donor site (SD) were used to retain the RNA in the nucleus as to generate Rev-dependent mRNAs [23], [24]. Various combinations of these elements were inserted into the *Rluc* 3′ UTR restriction sites (XhoΙ and NotΙ in **Fig. 1A**). In addition, one of a let-7 binding sites was inserted into the PmeΙ restriction site. These vectors presented significantly diminished *Rluc* activity when compared to control firefly luciferase activity (**Fig. 1C**, Vectors 1–10 in the presence of pcDNA). When Rev-HA was coexpressed, luciferase activity increased in cells transfected with the empty vector or BulgeMut vectors carrying a correctly oriented RRE (Vectors 1, 2, 9 and 10, red arrows), which suggested that the mRNA was efficiently transported to the cytoplasm and translated (see also **Fig. S1B**, Vectors 3 and 4; **Fig. S1C**, Vectors 3 and 4, red arrows). To assess the ability of Rev to export these mRNAs into the cytoplasm, the cytoplasmic *Rluc* RNA levels were compared in the presence of pcDNA and in the presence of Rev-HA. This analysis indicated that the cytoplasmic glyceraldehyde-3-phosphate dehydrogenase (G3PDH) and firefly luciferase mRNAs were enriched. In contrast, U1 snRNAs were enriched in the nucleus (**Fig. S2A–C**). In agreement with the *Rluc* assay results, the cytoplasmic *Rluc* RNA level was significantly reduced when pcDNA was coexpressed, suggesting the sequestration and degradation of these mRNAs in the nucleus. In contrast, the *Rluc* RNA levels increased to the levels of the psiCHECK control in the presence of Rev-HA (**Fig. S2D**, Vectors 1, 2, 9 and 10, red arrows). These results suggested that Rev transported these RRE-containing mRNAs; however, the increase in the level of transported mRNAs did not exceed the levels observed in the psiCHECK control, which suggested that further upregulation of the observed *Rluc* activities (Vectors 1, 2, 9 and 10 in **Fig. 1C**, red arrows) was dependent on translational-level Rev function. In contrast, the luciferase activity in cells transfected with the Bulge-, 3×Bulge- or Perfect-containing constructs was silenced regardless of the orientation of the RRE (**Fig. 1C**, Vectors 3–8, red arrowheads; **Fig. S1C**, Vectors 1 and 2, red arrowheads). These results suggested that the siRNAs and miRNAs interfered with target mRNA expression regardless of Rev activity. Similar results were also observed in the presence or absence of an SD (**Fig. S1D** and **E**), which was consistent with RRE regulation of nuclear sequestration of HIV-1 mRNAs [25]. Similar results were obtained when untagged Rev was coexpressed instead of Rev-HA (**Fig. S3A** and **B**).

To examine the silencing effects on unspliced RNAs, the chimeric intron downstream of the SV40 promoter was removed (**Fig. 1D**, pSVEP). The *Rluc* activities associated with BulgeMut-containing RNAs significantly increased in the presence of Rev-HA (**Fig. 1E**, Vectors 5 and 6, red arrows) compared to their intron-containing counterparts (**Fig. 1C**, Vectors 9 and 10, red arrows). Importantly, and in contrast to cells transfected with the psiCHECK constructs (**Fig. 1E**, Vectors 1 and 2, red arrowheads), the luciferase expression from Bulge-containing RNAs in cells transfected with pSVEP-derived vectors was not silenced, regardless of the location of the RRE relative to the Bulge site (Vectors 3 and 4, blue arrows). Moreover, the constructs that lacked the enhancer (pSVP) presented similar, but lower in magnitude, effects on these activities (Vectors 7–10, red and blue arrows).

**Figure 2:**
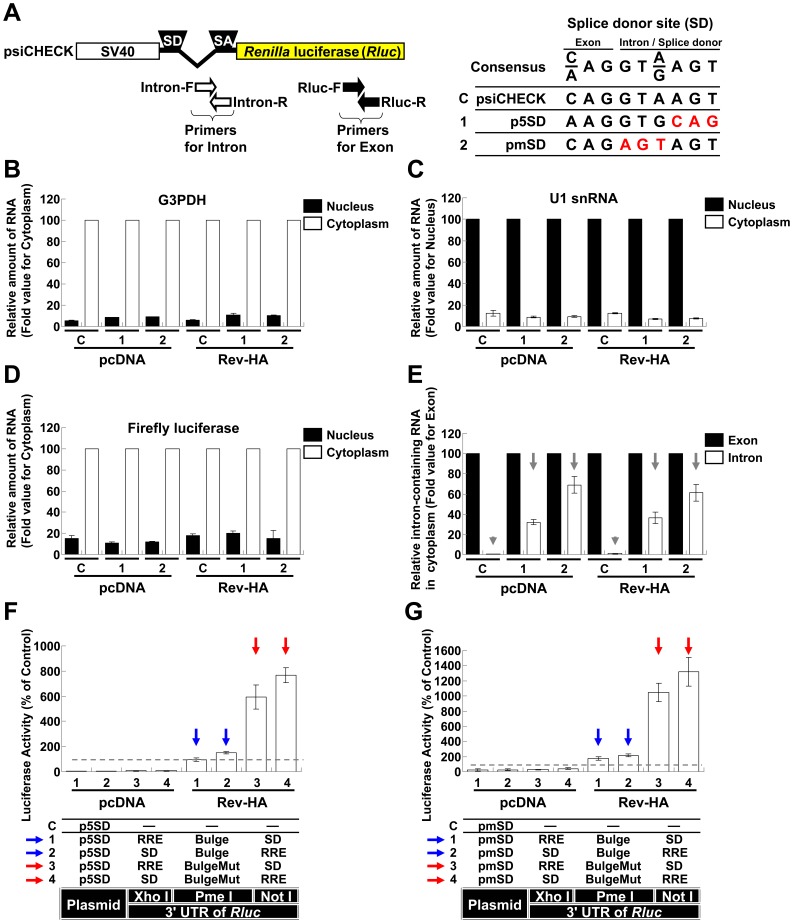
Analysis of Rev-dependent export on RNAs with mutations in splice donor site. (A) A schematic of the psiCHECK-2 vector (psiCHECK) in which the locations of primers to amplify the intron region or exon region of the *Rluc* are illustrated. The splice donor and the juxtaposed exon sequences (SD) are shown. The sequence mutated from the psiCHECK vector is shown in red. The p5SD vector was exchanged with the fifth SD (6,722–6,730) of pNL4-3. The pmSD has mutations in the highly conserved SD sequences. (B) The nuclear and cytoplasmic level of G3PDH RNA in HeLa cells was analyzed by RT-qPCR. The cytoplasmic level was set to 100. (C) The nuclear and cytoplasmic level of U1 snRNA. The nuclear level was set to 100. (D) The nuclear and cytoplasmic level of firefly luciferase RNA in which the cytoplasmic level was set to 100. (E) The levels of *Rluc* RNAs transported into the cytoplasm were analyzed by RT-qPCR. The intron region and exon region were amplified using the primers shown in (A). The RNA levels amplified by primers in the exon region were set to 100. The gray arrowhead and arrow point to the intron-containing *Rluc* RNA levels in each transfected cell. (F) The let-7-mediated silencing of Rev-HA-exported RNAs from p5SD. The red arrow points to the vectors that presented altered *Rluc* activity in the presence of Rev-HA. The blue arrow points to the Bulge-containing constructs that carry a correctly oriented RRE and were not silenced in the presence of Rev-HA. (G) The let-7-mediated silencing of Rev-HA-exported RNAs from pmSD. The *Renilla*/firefly luciferase value was assessed, and the data presented are the mean ± S.D. of the percentage normalized to the empty vector C.

Among other mechanisms, HIV-1 utilizes a suboptimal SD to produce unspliced RNAs [26]. The psiCHECK SD corresponds to the consensus sequence (**Fig. 2A**). When the cells that had been transfected with psiCHECK were fractionated into their nuclear and cytoplasmic fractions and we assessed the expression levels of the *Rluc* RNAs in the cytoplasm using intron- and exon-specific primers, the intron-containing RNAs were rarely detected. This result suggested that complete splicing occurred before export to the cytoplasm (**Fig. 2E**, Vector C, gray arrowheads). The integrity of the nucleus and RNA appeared to be preserved (**Fig. 2B–D**). To reduce splicing, the psiCHECK SD was replaced with an NL4-3 fifth SD (**Fig. 2A**, p5SD). To promote further abrogation of the splicing, the highly conserved sequence was mutated (**Fig. 2A**, pmSD). These modified vectors expressed more intron-containing RNAs in the cytoplasm of the transfected cells than the psiCHECK vector (**Fig. 2E**, Vectors 1 and 2, gray arrows). The *Rluc* activities associated with BulgeMut-containing RNAs significantly increased in the presence of Rev-HA, similar to the pSVEP experiments (**Fig. 2F and G**, Vectors 3 and 4, red arrows; **Fig. 1E**, red arrows). In addition, luciferase expression associated with Bulge-containing RNAs was not silenced (**Fig. 2F and G**, Vectors 1 and 2, blue arrows). These results suggested that unspliced RNAs were efficiently expressed and appeared to override miRNA-mediated interference following Rev-mediated transport.

### HIV-1 LTR-driven mRNA Modulation Influence on miRNA-driven Suppression Avoidance

**Figure 3:**
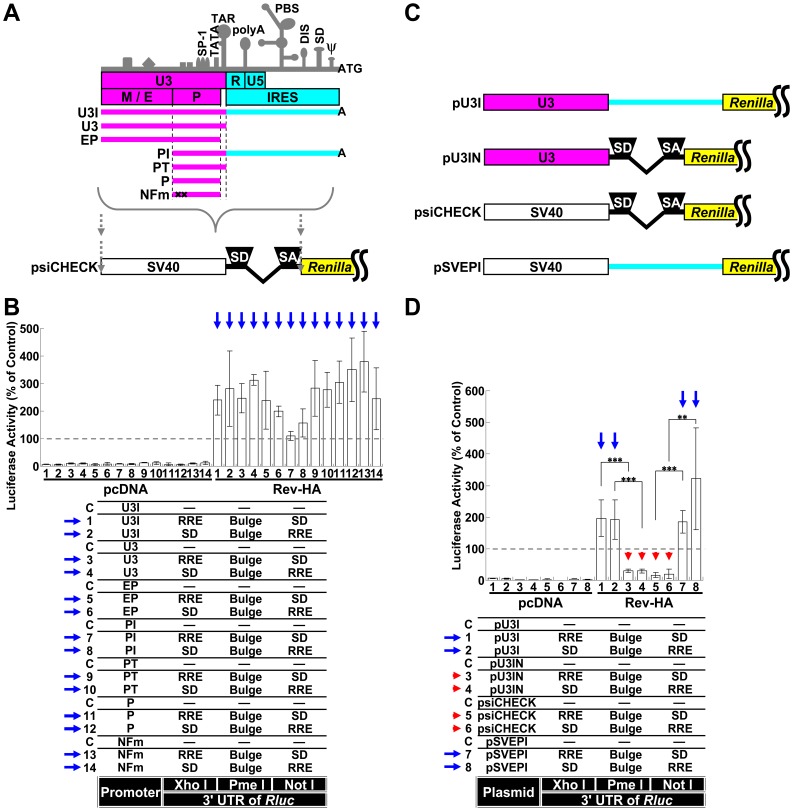
Silencing of RNAs transcribed from the HIV-1 LTR. (A) A schematic of the HIV-1 LTR. The important transcriptional elements in the U3 region, characteristic secondary structures and major functional regions are shown. “M/E” indicates the modulator and enhancer regions, and “P” indicates the promoter region. Several truncated promoters, U3I, U3, EP, PI, PT, P and NFm, which is identical to P but carries mutations in the two NF-κB binding sites that render them nonfunctional, were generated as shown. The SV40 promoter and intron region of the psiCHECK were replaced with various regions of the LTR. (B) The RNAs transcribed by promoters derived from several regions of the HIV-1 LTR were exported by Rev-HA and analyzed to determine the effects of miRNA-mediated interference. The blue arrow points to the Bulge-containing constructs that carry a correctly oriented RRE and were not silenced in the presence of Rev-HA. (C) The schematics of the pU3I, pU3IN, psiCHECK and pSVEPI constructs. In the pU3IN and pSVEPI constructs, the region downstream of the TAR (light blue region) of pU3I and the region containing the chimeric intron from the psiCHECK were exchanged with each other. (D) The effects of splicing on miRNA-mediated silencing were analyzed using chimeric promoters derived from the LTR and SV40 promoters. The red arrowhead points to the Bulge-containing constructs that carry an intron and a correctly oriented RRE and were silenced in the presence of Rev-HA. The blue arrow points to the Bulge-containing constructs without an intron that carry a correctly oriented RRE and were not silenced in the presence of Rev-HA. The *Renilla*/firefly luciferase value was assessed in each graph. The empty vector C was used as a control in each promoter setting, and the results are presented as the mean ± S.D. as a percentage of the control from three independent experiments. “pcDNA” denotes the pcDNA3.1(+) plasmid. ***P<0.001, **P<0.005.

To better mimic the physiological virus transcription system, the SV40 promoter and psiCHECK intron region were replaced with various portions of the HIV-1 long terminal repeat (LTR **Fig. 3A**). RNAs containing the Bulge sequence but expressed from these LTR-driven constructs were not silenced (**Fig. 3B**, blue arrows). These results were similar to the results observed with the vector that possessed an SV40 promoter without an intron (**Fig. 1E**, blue arrows). The vector required only the core promoter region for robust gene expression and did not require NF-κB binding sites (“P” and “NFm” in **Fig. 3A and B**, Vectors 11–14). The downstream U3 region of the LTR (light blue region in **Fig. 3A and C**), which may function as an internal ribosome entry site (IRES) [27], was replaced by the psiCHECK intron (**Fig. 3C**, pU3IN), and the psiCHECK intron was replaced by the IRES region of the LTR (pSVEPI). When this construct was tested, miRNA-mediated suppression was confined to spliced RNAs (pU3IN and psiCHECK in **Fig. 3C and D**, Vectors 3–6, red arrowheads; the blue arrows point to the constructs without an intron that were not silenced in the presence of Rev-HA).

However, the reversal of silencing for RNAs containing 3×Bulge was less efficient than 1×Bulge-containing RNAs (**Fig. S4A**, Vectors 3 and 4, dashed blue arrows), and RNAs containing Perfect sequences were not very effective at silencing (Vectors 5 and 6, blue arrowheads). Therefore, the replication of HIV-1 could be repressed by several miRNAs that targeted the mRNA or by the exogenous overexpression of an siRNA. However, the coexpression of the potential modulators of HIV-1 gene expression and replication Tat, Vpr and APOBEC3G also appeared to modulate the observed silencing effects for 3×Bulge-containing RNAs (**Fig. S4B**). Although U3-driven RNAs that contained only a Bulge or a Perfect sequence in the *Rluc* 3′ UTR were affected by cotransfection of these vectors (**Fig. S4C**, Vectors 1 and 3), Rev-dependent RNA appeared to be more affected [28]–[31]. Therefore, Rev-mediated RNAs appeared to be modulated in several ways in HIV-1-infected cells. However, the effect of Vpr was not dependent on IRES function because the U3-driven promoter did not contain this region (light blue region in **Fig. 3A and C**).

### The Identification of Potential miRNA Target Sequences in the HIV-1 Genome

**Figure 4:**
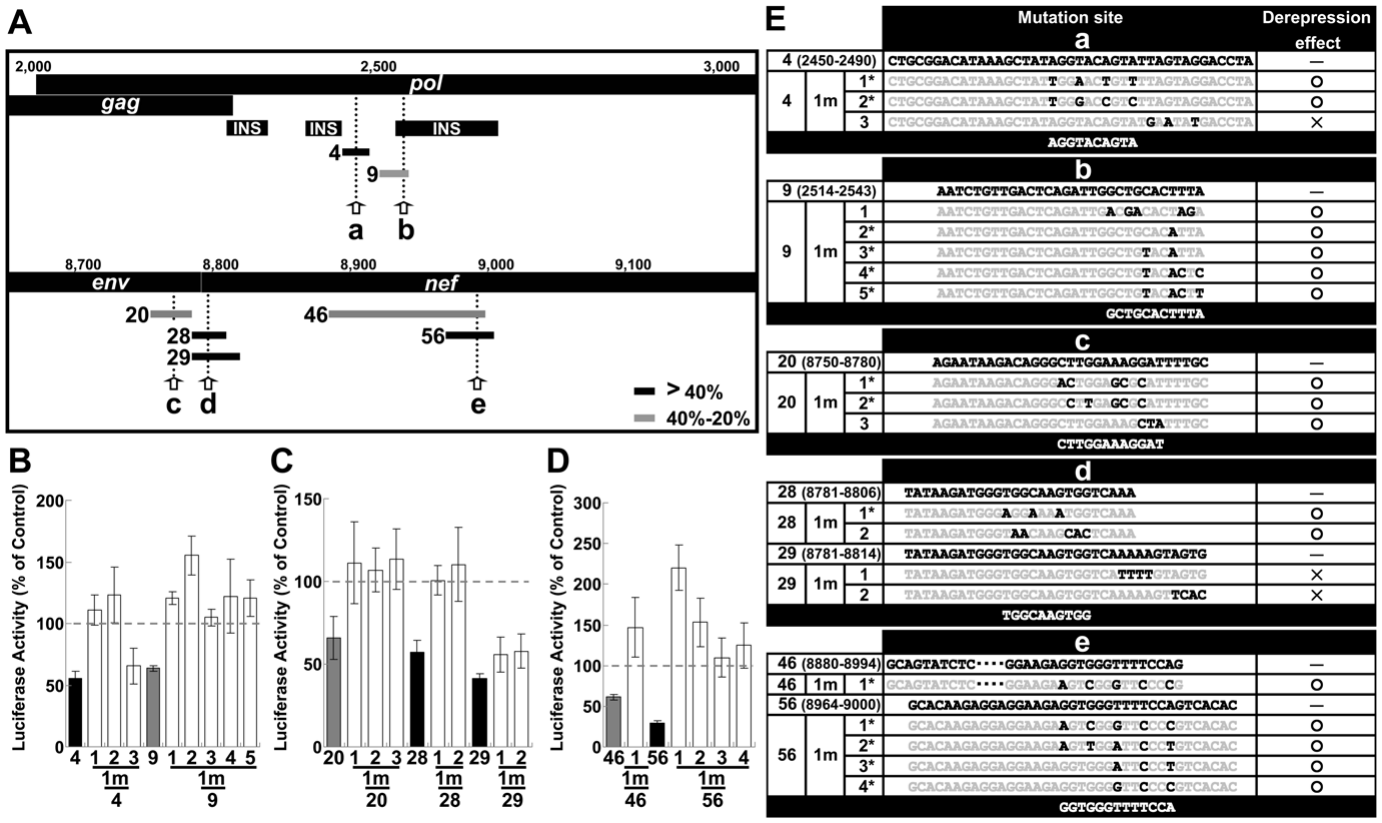
The effects of mutations in suppressive sequences in the *pol* and *env*-*nef* regions. (A) The *pol* and *env*-*nef* regions of pNL4-3 are illustrated. The *cis*-acting instability elements (INS) are also illustrated below the genome. The arrows indicate the mutated sites in the *pol* (sites “a” and “b”) and *env*-*nef* (sites “c”, “d” and “e”) regions. The black (>40%) and gray (40–20%) bars represent the repressed region. The number and bar pattern corresponds to the graph below. (B) The effects of mutations introduced into the repressive sequences in the *pol* region (sites “a” and “b”) were assessed in Jurkat cells. The “1 m” indicates the vector that has the mutated sequence introduced into the repressive site. (C) Mutational assay of the suppressive sequences (sites “c” and “d”) in the *env*-*nef* region. (D) Mutational assay of the suppressive sequences (site “e”) in the *nef* region. The *Rluc* activity was normalized to the firefly luciferase activity, and the data shown are the mean ± S.D. of the percentage of the activity of the empty psiCHECK-2 vector (psiCHECK). (E) Pattern mutations and the effects of each dissected sequence in the *pol* (sites “a” and “b”) and *env*-*nef* (sites “c”, “d” and “e”) regions. The gray characters represent unchanged residues. Asterisks denote mutations that did not change any amino acids (see Table S1 for details).

**Figure 5:**
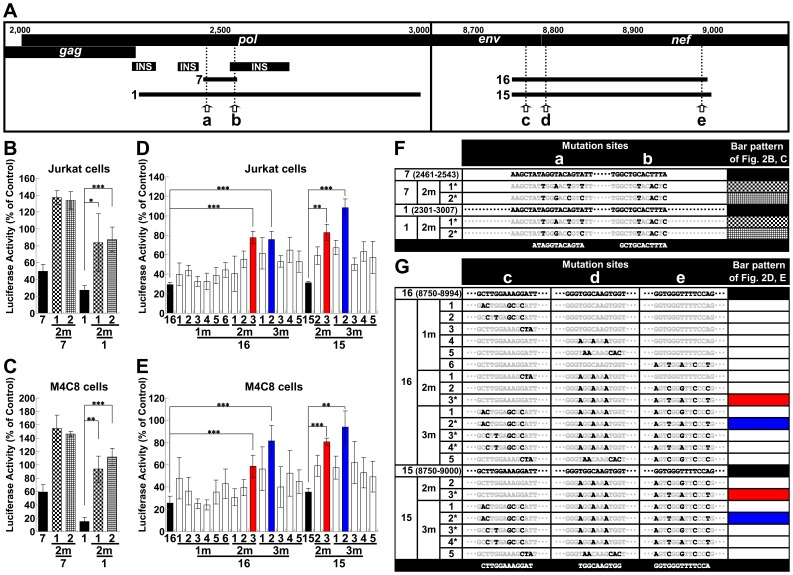
The effects of combining multiple mutations in the *pol* and *env*-*nef* silencing regions. (A) The *pol* and *env*-*nef* regions of pNL4-3 and the position of the *cis*-acting instability elements (INS) are illustrated. The arrows indicate the mutated sites in the *pol* (sites “a” and “b”) and *env*-*nef* (sites “c”, “d” and “e”) regions. The number and bar pattern corresponds to the graph below. (B) The effects of multiple mutations in the *pol* region (sites “a” and “b”; elements 1 and 7) were assessed in Jurkat cells. Six independent experiments were performed. The bar patterns correspond to the mutational patterns shown in (F). The “2 m” indicates that the vector has two mutation sites in the repressive sequence. (C) The effects of multiple mutations in the *pol* region were assessed in M4C8 cells. (D) The effects of multiple mutations introduced into the *env*-*nef* region (sites “c”, “d” and “e”; elements 15 and 16) were assessed in Jurkat cells. Four independent experiments were performed. The bar patterns correspond to the mutational patterns shown in (G). The “1 m”, “2 m” and “3 m” indicate that the vector has one, two and three mutation sites individually. The red and blue bars indicate the more derepressed mutational patterns. (E) The effects of multiple mutations introduced into the *env*-*nef* region were assessed in M4C8 cells. The psiCHECK was used as a control, and the results are expressed as the mean ± S.D. as a percentage of the control. ***P<0.001, **P<0.005 and *P<0.05. (F) Combinations of mutated patterns in the *pol* region and the bar patterns in graph B and C. (G) Combinations of mutated patterns in the *env*-*nef* region and the bar patterns in graph D and E. The gray characters represent unchanged residues. The asterisk denotes the mutational pattern used for the generation of mutant viruses.

**Figure 6:**
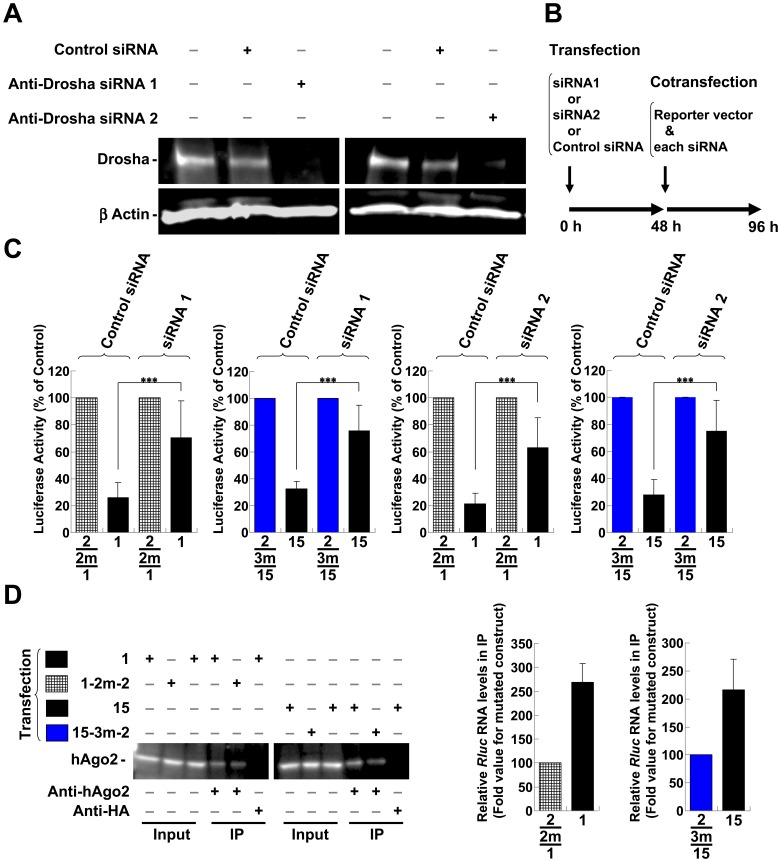
The characterization of suppressive sequences in the *pol* and *env*-*nef* regions. (A) The effect of each anti-Drosha siRNA (siRNA1 and siRNA2) on Drosha protein level was evaluated by western blot using an anti-Drosha antibody. The reduced amount of Drosha protein in the Jurkat cells was confirmed at 48 h after treatment with anti-Drosha siRNAs compared with the control or mock treatment. After stripping, the membrane was reprobed with an anti-ß actin antibody. (B) Schematic representation of the experimental procedures for RNAi experiments in Jurkat cells. (C) The effects of the anti-Drosha siRNA at the repressed “a ” and “b” sites in the *pol* region (element 1 in **Fig.** **5A**) and sites “c”, “d” and “e” in the *env*-*nef* region (element 15 in **Fig. 5A**) were assessed in Jurkat cells (black bars). The derepressive effect was confirmed in each region relative to the corresponding vectors with mutations in the repressive sites (mutation 1-2m-2 in the *pol* region (lattice bars) and 15-3m-2 in the *env*-*nef* region (blue bars); see also **Fig. 5B**–**G**). The bar patterns correspond to the mutational patterns shown in **Fig. 5**. The *Renilla*/firefly luciferase value was assessed, and the data shown are the mean percentages ± S.D. of the mutated vector from six independent experiments. ***P<0.001. (D) The Jurkat cells were transfected with each vector, and RNA-immunoprecipitation (IP) using anti-human Ago2 (hAgo2) antibody was performed. The input and the IP samples were adjusted equal volume and the same volume was loaded and analyzed by western blot. The levels of the immunoprecipitated *Renilla* luciferase mRNAs compared to Firefly luciferase mRNAs produced from the vectors (Vectors 1 and 15) and the mutated vectors (Vectors 1-2m-2 and 15-3m-2) were analyzed by RT-qPCR. The normalized values of the *Renilla*/firefly luciferase levels are shown. The each mutated vector was set to 100.

The observed Rev-mediating effect caused by miRNA interference implied a defensive mechanism against host miRNAs during HIV-1 replication. An analysis of the suppressive sequences within the HIV-1 genome would provide further insight into the relationship between HIV-1 and cellular miRNAs during HIV-1 replication. To identify the regions of HIV-1 that could be targeted by miRNAs during replication, several regions from the HIV-1 genome were inserted into the *Renilla* luciferase (*Rluc*) gene downstream of the stop codon in a psiCHECK-2 vector (psiCHECK). The resulting constructs were transfected into T cell-derived Jurkat, Clone E6-1 (Jurkat) and Molt-4, Clone 8 (M4C8) cells. This strategy has been widely utilized to validate the authenticity of miRNA target sites and their repression susceptibility [32]. Furthermore, this strategy allows for the identification of novel small RNAs with biological activities based on the inferred effects of the small RNAs on RNA degradation or translational impediment [33], [34]. Several potential suppressive regions were identified in both Jurkat and M4C8 cells. We examined these regions sequentially and identified several suppressive sequences, a number of which were characterized further; however, several of the identified sites were not fully characterized or examined in detail in this study (**Fig. S5A** and **B**). Several approximately 30-base suppressive sequences were identified in the *pol* and *env*-*nef* regions (**Fig. 4A–D**, black and gray bars; see also **Fig.** **S6A** and **B** for other regions). Site-directed mutagenesis of the sensitive sites in the *pol* region (sites “a” and “b”) and in the *env*-*nef* region (sites “c”, “d” and “e”) was used to generate sequences that ameliorated silencing (**Fig. 4B–E**, “1 m” indicates the vector with the mutation). It is important to note that the amelioration was achieved without affecting the amino acid sequence (**Fig. 4E**, asterisks; see **Table S1A** and **B** for details). In addition, the expression of HIV-1 proteins is known to be restricted by *cis*-acting instability elements (INS) or cis-acting repressive sequences (CRS) in addition to adverse codon bias, which is reflected in the high A/U content of the HIV-1 genome [35], [36]. Therefore, the suggested and predicted INS/CRS regions were compared to the identified regions, and mutation site “b” was included in the previously suggested INS3 (**Fig. 4A**) [37]. However, we obtained a significant derepression associated with the “b” site mutations through the introduction of a 1-base T-to-A mutation and a 2-base mutation from CT to TA, which resulted in an increase in the AU content (**Fig. 4B and E**, 9-1m-2 and 9-1m-3). These mutations contrasted with previously identified clustered point mutations in the AU-rich sequence [37]. Therefore, it was unlikely that the suppressive effect of the “b” site was related to the INS/CRS effect. Nevertheless, it is possible that this site played dual roles. Several mutations were assessed in combination to confirm whether each mutation examined in the dissected regions was effective or sufficient to relieve silencing. The suppressive sequence (**Fig. 5A**, elements 1 and 7) in the *pol* region was effectively disabled in both cell lines by introducing two point mutations (**Fig. 5B, C and F**, “2m” indicates the vector with two mutation sites). These results also suggested that the expression vectors were not significantly affected by INS, which cover some of the element 1 region (**Fig. 5A**). However, considering the greater efficiency of *Rluc* activity observed with element 7 mutants (7-2m-1 and 7-2m-2) over element l mutants (1-2m-1 and 1-2m-2), the INS might have moderately modulated the effect. A significant reduction in suppression was also observed upon *env*-*nef* region mutation. However, there was a minor difference in silencing that was dependent on the applied length (**Fig. 5A**, between elements 15 and 16 in the *env*-*nef* region). The degree of derepression increased in proportion to the number of sites mutated; however, the effect of each mutation varied (**Fig. 5D, E and G**, “1m ”, “2 m” and “3 m” indicate the vectors with one, two and three mutation sites, respectively). A significant reduction was observed for two- (red bars, 15-2m-3 and 16-2m-3) and three-base mutations (blue bars, 15-3m-2 and 16-3m-2). The differences in the degree of derepression might have been due to the position of the target site in the 3′ UTR, the surrounding sequence in the 3′ UTR, the overall mRNA structure or the binding of regulatory proteins [38]–[41]. Nevertheless, a more prominent effect was observed when three copies of one repression site (site “d” in the *env*-*nef* region) were concatenated (**Fig. S7**, 3×28). Such concatenation has previously been shown to synergistically enhance the effect of the miRNA [42]. To address whether these silenced sites are directly targeted by miRNAs, miRNA generation was reduced by transfecting synthetic anti-Drosha siRNAs into the cells [43], [44], and the derepressive effect of these silencing sites was assessed. The knockdown in Jurkat cells was confirmed by western blot 48 h after treatment with anti-Drosha siRNAs (**Fig. 6A**). Therefore, the constructs bearing native sequences at the “a” and “b” sites in the *pol* region (**Fig. 5A**, element 1) or sites “c”, “d” and “e” in the *env*-*nef* region (**Fig. 5A**, element 15) were transfected and the derepression at 48 h post-transfection was assessed. However, the observed derepressive effects were marginal, and therefore, the vector and the anti-Drosha siRNAs were cotransfected using the procedure shown in **Fig. 6B**. The constructs bearing suppressive sequences were significantly derepressed (**Fig. 6C**, Vectors 1 and 15, siRNA1 and siRNA2 treatment, black bars) compared with the mutated versions (1-2m-2, lattice bars and 15-3m-2, blue bars). These results suggested that these sites were regulated by Drosha-produced miRNAs. The same vectors were also compared via ribonucleoprotein immunoprecipitation (IP) assay using anti-human Ago2 (hAGO2) antibody. The hAGO2 was pulled-down by anti-hAGO2 antibody but not by an unrelated anti-HA antibody (**Fig. 6D**). In addition, the enrichment of the coprecipitated *Rluc* RNAs appeared to be preferred for the repressed vectors (Vectors 1 and 15, black bars). These results suggested that the suppressive sites appeared to be accessible to canonical miRNAs when inserted into a 3′ UTR [45] and also appeared to be controlled and silenced by miRNAs in the context of the *pol* and *env*-*nef* regions. Taken together, the results indicated that the potential miRNA targets in the HIV-1 genome could be identified by miRNA profiling in Jurkat cells with the prediction of the seed sequence and with the assumption that the miRNA:mRNA binding site would be included in the mutated sites that were depreressed (**Table 1**). Many of these predicted miRNAs have also been suggested to operate in primary T cells [18], [19]; however, the exact contribution of these miRNAs to cellular regulation is currently unknown, and the miRNAs whose binding are contributed by the 3′ region or truncated miRNAs are excluded from this list. In addition, an alternation in the RNA binding protein to aid RISC function or structural changes to the RNA by mutations are also a possibility [38]-[41]. When we analyzed these sites in HeLa cells and 293T cells, which are conventionally used in HIV-1 analysis, we observed similar patterns of repression and derepression for a subset of the confirmed mutations (**Fig. S8A** and **B**). Therefore, these sites appeared to be widely repressed in multiple cell types, and it is possible that the identified sites were preferentially accessed or targeted by several miRNAs, potentially with similar seed sequence compositions. The effects of these miRNAs were dependent on their cellular concentrations.

**Table 1:** Putative microRNAs predicted to target *pol* or *env*-*nef* region.

Site	Target Sequence	Name of miRNA	Free energy	Relative amount §	Primary T cells
a	ATCTGCGGACATAAAGCTATAGGTACAGTATTAGTAGGACCTACACCTGT	hsa-miR-200c	−18.7	43.5	+
a	ATCTGCGGACATAAAGCTATAGGTACAGTATTAGTAGGACCTACACCTGT	hsa-miR-636	−20.9	1.7	+
b	TAATTGGAAGAAATCTGTTGACTCAGATTGGCTGCACTTTAAATTTTCCC	hsa-miR-17	−22.4	12617.5	+
b	TAATTGGAAGAAATCTGTTGACTCAGATTGGCTGCACTTTAAATTTTCCC	hsa-miR-20b	−21.6	1016.6	+
b	TAATTGGAAGAAATCTGTTGACTCAGATTGGCTGCACTTTAAATTTTCCC	hsa-miR-93	−22.1	1477.8	+
b	TAATTGGAAGAAATCTGTTGACTCAGATTGGCTGCACTTTAAATTTTCCC	hsa-miR-106a	−22.4	17542.1	+
b	TAATTGGAAGAAATCTGTTGACTCAGATTGGCTGCACTTTAAATTTTCCC	hsa-miR-106b	−23.5	435.9	+
c	CTATTCGCCACATACCTAGAAGAATAAGACAGGGCTTGGAAAGGATTTTG	hsa-miR-362-5p	−22.9	21.1	+
c	CTATTCGCCACATACCTAGAAGAATAAGACAGGGCTTGGAAAGGATTTTG	hsa-miR-378*	−31.2	5.5	+
c	CTATTCGCCACATACCTAGAAGAATAAGACAGGGCTTGGAAAGGATTTTG	hsa-miR-500	−20	17.1	+
c	CTATTCGCCACATACCTAGAAGAATAAGACAGGGCTTGGAAAGGATTTTG	hsa-miR-501-5p	−23.1	4.8	
D	GAAAGGATTTTGCTATAAGATGGGTGGCAAGTGGTCAAAAAGTAGTGTGA	hsa-miR-193b	−20.8	69.2	+
D	GAAAGGATTTTGCTATAAGATGGGTGGCAAGTGGTCAAAAAGTAGTGTGA	hsa-miR-221	−20.6	83.3	+
D	GAAAGGATTTTGCTATAAGATGGGTGGCAAGTGGTCAAAAAGTAGTGTGA	hsa-miR-328	−23.2	17	+
D	GAAAGGATTTTGCTATAAGATGGGTGGCAAGTGGTCAAAAAGTAGTGTGA	hsa-miR-500	−26	17.1	+
D	GAAAGGATTTTGCTATAAGATGGGTGGCAAGTGGTCAAAAAGTAGTGTGA	hsa-miR-502-5p	−21.2	1.34	+
D	GAAAGGATTTTGCTATAAGATGGGTGGCAAGTGGTCAAAAAGTAGTGTGA	hsa-miR-660	−22.8	86.2	
E	AGCACAAGAGGAGGAAGAGGTGGGTTTTCCAGTCACACCTCAGGTACCTT	hsa-miR-7	−20.4	13.2	+
E	AGCACAAGAGGAGGAAGAGGTGGGTTTTCCAGTCACACCTCAGGTACCTT	hsa-miR-185	−21.8	19.2	+
E	AGCACAAGAGGAGGAAGAGGTGGGTTTTCCAGTCACACCTCAGGTACCTT	hsa-miR-491-5p	−23.9	6.8	+
E	AGCACAAGAGGAGGAAGAGGTGGGTTTTCCAGTCACACCTCAGGTACCTT	hsa-miR-550	−30	1.2	

§ Fold value for RUN6B.

The microRNA binding site is underlined. The miRNAs suggested to be present in primary T cells or HIV-1-infected primary T cells are shown as “+” in the “Primary T cells” column [18], [19].

### The Effects of Mutating miRNA Target Sites on Virus Replication

**Figure 7:**
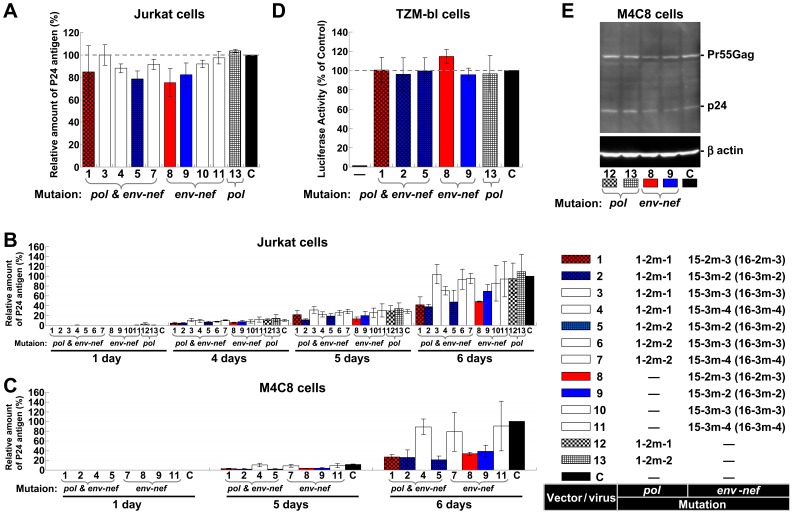
The effects of the mutations on viral replication and Gag processing. (A) pNL4-3 plasmids with mutations in *pol, env*-*nef* or both were constructed (marked with asterisks in **Fig. 5F** and **G**). Jurkat cells were transfected with the resulting plasmids or pNL4-3 (“C”). The amount of p24 produced at 24 h after transfection was analyzed. The amount of p24 produced by the pNL4-3-transfected cells at 24 h post-transfection was set at 100%, and the p24 production in each tested line was expressed as the mean ± S.D. percent of this amount. The bar patterns correspond to the mutational patterns shown in the bottom right and in **Fig. 5**. (B) Mutant viruses or NL4-3 (“C”) were obtained by transfecting 293T cells with each plasmid, and the resulting viruses were normalized based on the amount of p24. Jurkat cells were infected with 100 ng of p24-normalized virus. The amount of p24 produced by NL4-3-infected cells at 6 days post-infection was set at 100%, and each p24 amount was expressed as the mean ± S.D. percent of this amount. (C) M4C8 cells were infected with 10 ng of p24-normalized virus and analyzed as described in (B). (D) TZM-bl cells were infected with 10 ng of p24-normalized virus. The firefly luciferase activities were assessed at 40 h post-infection and the luciferase activity by NL4-3-infected cells was set at 100%. The (−) indicates the activity of the mock-treated cells. (E) M4C8 cells were infected with 10 ng of p24-normalized virus. The amount and processing of Gag in the infected cells were analyzed at 5 days post-infection by western blot using the HIV-1 p24 Gag monoclonal antibody. The membrane was then stripped and reprobed with an anti-ß actin antibody as a loading control.

Combinatorial site-directed mutagenesis was performed on the HIV-1 genome to determine whether these sites were readily accessible and, if so, what impact mutating these regions had on HIV-1 replication. Several sets of mutations were selected based on their derepressive effects (**Fig. 5F and G**, asterisks). Importantly, amino acid changes and rare codon usage were avoided. When these vectors were transfected into Jurkat cells, a modest reduction in the amount of virus produced at 24 h post-transfection was observed (**Fig. 7A**; bar pattern corresponds to the mutation pattern in the bottom right) compared with cells that had been transfected with pNL4-3 (“C”). To quantitate this reduction, the Jurkat cells were infected with 100 ng of ***p24***-***normalized*** virus, and virus production was monitored every day after infection (**Fig. 7B**). The level of virus production did not change significantly following infection with viruses carrying mutations in the *pol* region (Viruses 12 and 13, check and lattice bars) or the less derepressive mutations in the *env*-*nef* region (Viruses 10 and 11, white bars). However, decreased levels of virus production were observed following infection with viruses carrying the more derepressive combinatorial mutations in the *env*-*nef* region (Viruses 8 and 9, red and blue bars). When these mutations were combined with those in the *pol* region, a slightly enhanced effect was observed (Viruses 1, 2 and 5). When M4C8 cells, which are more susceptible to HIV-1 infection, were infected with 10 ng of p24-normalized virus, a more profound effect was observed (**Fig. 7C**, Viruses 1, 2, 5, 8 and 9). To ensure that the integrity of these mutant viruses was preserved, the viruses that reduced infectivity were compared to unaltered and NL4-3 viruses using a TZM-bl assay [46], [47]. Briefly, TZM-bl cells are HeLa cell derivatives that express high levels of CD4 and both co-receptors CXCR4 and CCR5. The cells can be stably transduced with LTR-driven firefly luciferase. Challenging these indicator cells with NL4-3 and mutant viruses results in the induction of luciferase, which allows facile detection of infection and titration of the amount of infected virus. When the luciferase readout was examined by infecting TZM-bl cells with the equivalent of 0, 0.5, 2.5, 10 and 20 ng of p24, the linear range of the activity was assessed using 0.5, 2.5, 5 and 10 ng of p24 (data not shown). Then, the infectivity per 10 ng of p24 was compared. The results indicated comparable levels of luciferase activity among the virus stocks analyzed, and mock treated cells (−) did not present any significant activity (**Fig. 7D**). In addition, Gag processing in the M4C8 cells at 5 days post-infection was analyzed using an anti-p24 Gag monoclonal antibody. Lysates from cells infected with the mutant viruses were not significantly different from those of cells infected with NL4-3. These results were consistent with the sequence analysis (**Fig. 7E**). However, mutations in the *pol* region appeared to enhance the variability of virus production; that is, the amount of virus produced was up or downregulated more severely. The results shown are examples of unchanged viral production (Viruses 12 and 13, check and lattice bars). In contrast, the production of Gag and processed proteins appeared to be reduced in cells infected with viruses bearing mutations in the *env*-*nef* region (Viruses 8 and 9, red and blue bars). The NL4-3 activity level reduction was approximately 40% to 65% of NL4-3, reflective of reduced virus replication; however, the possibility of a specific effect on the production or stability of Gag proteins remains.

### The Effect of Rev-dependent Transport on the Suppressive Regions within HIV-1

**Figure 8:**
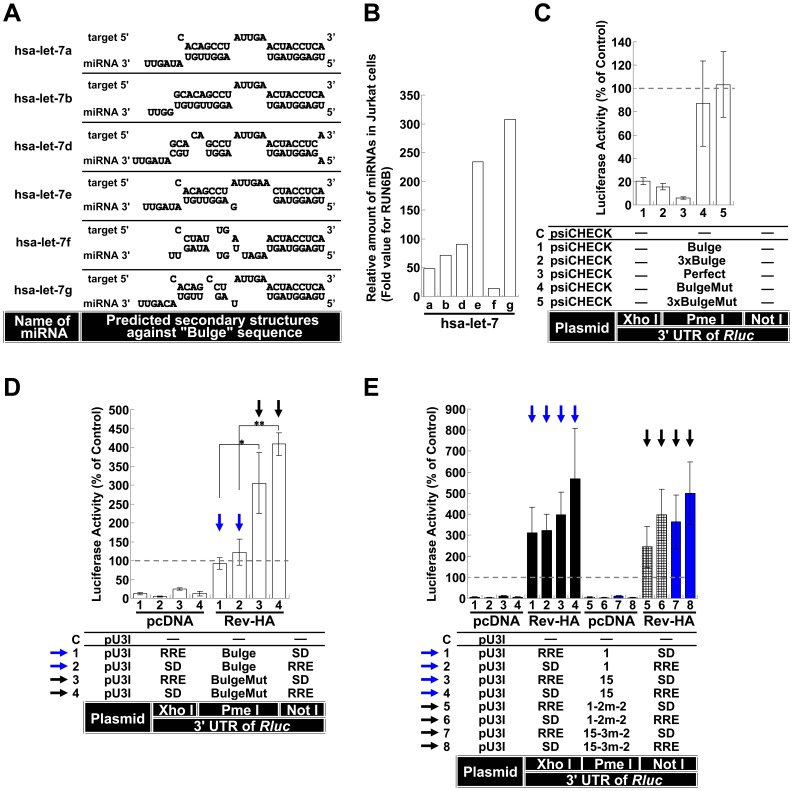
The effect of Rev-dependent export for Let-7 family members and suppressive sequences within HIV-1 in Jurkat cells. (A) The secondary structure of the miRNA/mRNA duplex of let-7 family members targeting the Bulge sequence was predicted using the RNA-hybrid program. (B) The relative RNA expression levels in the let-7 family members from Jurkat cells were calculated following normalization to the internal control RUN6B. (C) The silencing of RNAs containing the let-7 targeting-sequences in Jurkat cells. (D) The effects of the Bulge and BulgeMut sequences on RNA export by Rev-HA in Jurkat cells. The blue arrow points to the Bulge-containing constructs and the black arrow points to the BulgeMut-containing vectors. Three independent experiments were performed. **P<0.005, *P<0.05. (E) The effect of Rev-mediated export on RNAs in which the suppressive sequences identified in the *pol* (element 1 in **Fig. 5A**) and *env*-*nef* (element 15 in **Fig. 5A**) regions were inserted into the *Rluc* 3′ UTR was assessed in Jurkat cells (black bars, blue arrows). The corresponding vectors with mutations in the repressive sites (lattice and blue bars, black arrows) were also assessed as well (mutation 1-2m-2 in the *pol* region and 15-3m-2 in the *env*-*nef* region; **Fig. 5B–G**). The bar patterns correspond to the mutational patterns shown in **Fig. 5**. The *Renilla*/firefly luciferase value was assessed in each graph. The empty vector C was used as a control, and the results are presented as the mean ± S.D. as a percentage of the control. “pcDNA” denotes the pcDNA3.1(+) plasmid.

The effects of viruses bearing mutations in the suppressive sequences implied that these sites were accessible within the HIV-1 genome and were involved in virus production during replication. We set out to examine whether *pol-* and *env*-*nef*-dependent silencing could be overcome if the RNAs containing these sequences were exported through Rev-dependent transport. To examine whether the suppressive sequences could be mediated by Rev in Jurkat cells in an unspliced state as observed in HeLa cells, we first analyzed the let-7 target sequence. Because miRNA profiling revealed the presence of significant amounts of let-7 family members in Jurkat cells (**Fig. 8A and B**), we examined let-7 target sequences in Jurkat cells. The silencing effects of let-7 family members were also confirmed in Jurkat cells (**Fig. 8C**, Vectors 1–3). When the unspliced RNAs were transported by Rev, the silencing was completely blocked (**Fig. 8D**, Vectors 1 and 2, blue arrows), similar to what was observed in HeLa cells (**Fig. 3D**, Vectors 1 and 2, blue arrows). Because the *pol* and *env*-*nef* regions have several suppressive sites (sites “a” and “b” in the *pol* region and sites “c”, “d” and “e” in the *env*-*nef* region in **Figs. 4A** and **5A**), the entire lengths of these regions (**Fig. 5A**, elements 1 and 15) were inserted into the PmeΙ site of the *Rluc* 3′ UTR. When these vectors were cotransfected into Jurkat cells with the plasmid expressing Rev-HA, the silencing was completely blocked (**Fig. 8E**, Vectors 1–4, black bars and blue arrows). Specifically, the observed effects were not significantly altered in the wild-type sequence vectors with mutant *pol* and *env*-*nef* regions (Vectors 5–8, lattice, blue bars and black arrows). In addition, the difference was distinct from the difference observed with the Bulge- and BulgeMut-containing RNAs (**Fig. 8D and E**, blue arrows vs. black arrows).

### A Comparative Analysis of Rev and Rex Function and the Effects of Promoter type

A Rev-like protein is commonly used by more complex retroviruses. For example, the Rex protein of human T-cell leukemia virus type I (HTLV-1) can bind to the RRE of HIV-1, although its binding site is different from that of Rev [48]. Therefore, a Rex-expression plasmid, instead of a Rev-HA- or Rev-expression plasmid, was assessed. We found that, in the same way as Rev, Rex could also block let-7-mediated silencing for the vectors using pU3 (**Fig. S9A** and **B**, Vectors 1 and 2, blue arrows), and its effect was also splicing dependent (**Fig. S9A** and **B**, pU3IN, Vectors 3 and 4, red arrowheads). Therefore, functional conservation might exist, although the Rex activity was weak compared to Rev, even in the BulgeMut vector (**Fig. S9C**, Vectors 7 and 8, red arrows). In particular, Rex constructs did not present significant *Rluc* activities associated with the spliced RNAs when assessing psiCHECK-derived vectors. However, these differences appeared to exist. In addition to the weak Rex activity, the coexpression of Tat did not have an effect (**Fig. S9B**, Vectors 1 and 2 in the presence of Tat), and little inhibition was observed using the pU3 (HTLV) construct (Vectors 5 and 6, blue arrowheads). However, the coexpression of Rev-HA resulted in significant silencing inhibition and increased luciferase expression (**Fig. S9D**, Vectors 5 and 6, blue arrows). In this case, the coexpression of Tax (encoded by HTLV-1 and required for maximum transcriptional activation; **Fig. S9D**, Vector C in the presence of Tax) could not alleviate silencing (**Fig. S9B**, Vectors 5 and 6 in the presence of Tax). Thus, these results demonstrated a partial conservation between Rev and Rex. These results also suggested that the promoter type might influence their activities [49].

## Discussion

Rev has been suggested to mediate the effects of INS/CRS repressive sequences. Therefore, miRNA-mediated repressive sequences would suggest a wider repertoire of Rev functions. However, unspliced mRNA was required to augment expression and mediate miRNA silencing (**Fig. 1E**), which appears to be in contrast with the INS/CRS effect. In accordance with the previous findings, the insertion of an RRE that contained the INS/CRS facilitated the Rev-dependent augmentation of *Rluc* activity and counteracted the effect of an inhibitory 5′ splice site in the 3′ UTR regardless of splicing (**Figs. 1C, S** **1D** and **S3A**) [23], [25]. Nevertheless, considering the Rev-mediating effect on silencing activity for unspliced mRNAs, it would be reasonable to assume that longer sequences might be more susceptible to miRNA-mediated silencing. Alternatively, the unspliced RNA contains the major INS/CRS that are present in the *gag* and *pol* genes, which are removed from the partially spliced HIV-1 mRNA. Consequently, the unspliced RNA contains more features that promote Rev-dependent export and potentially stimulate its translation despite miRNA-mediated silencing. In fact, the presence of the INS/CRS element but not the AUUUA-instability element augmented Rev-dependent export when inserted with an RRE [50], and some protein complexes that have been found in the nucleus appear to be transported into and remain associated with the cytoplasm, presumably enhancing the activity of Rev-dependent RNA by stabilizing or increasing export or enhancing translation [51]–[54].

On the other hand, the identified suppressive regions in the HIV-1 genome were not silenced in the context of Rev-mediated export (**Fig. 8E**). Therefore, a change in the accessibility or the strength of the miRNA recognition of these sites during Rev-dependent transport is likely involved [55]. Importantly, the position of the miRNA target site, the surrounding sequence, the structure of the binding site and the regional binding of proteins all affect miRNA gene silencing [38]–[41]. In addition, the binding of an miRNA to its target site within the coding region as well as the 3′ UTR of the mRNA has also been suggested [56], [57]. Overall, at present, the full range of factors that contribute to miRNA binding to the target is not known, thus knowing the precise position of these susceptible sites within the HIV-1 genome is essential. In this regard, previously identified suppressive sites that had been suggested to contribute to maintaining latency were near the identified sites in the *env*-*nef* region, and both sites correspond to the 3′ region of the HIV-1 genome. However, the suggested sites did not show enough repression in the cell lines that were used in this study [18]. In contrast, the identified sites appear to be excluded from the previously suggested repressive sites. In light of this, the silencing in the *env*-*nef* region was observed in several cell lines; however, these silencing effects could be confined to cells that correspond to the activated state of CD4^+^ T cells. In fact, the silencing activity of the miRNAs that contribute to latency has been confined to lesting CD4^+^ T cells, and the sufficient repression has not been observed in the activated CD4^+^ T cells. On the other hand, the sites in the *pol* region had not prominent effects in mutational virus analysis, although some additive effects were observed in the combined mutations with the *env*-*nef* region. In addition, previous analysis has been limited in the 3′ region of the HIV-1 genome; therefore, it remains to be addressed whether this region has some influence in the resting state. However, the amounts of many miRNAs have been suggested to change differentially between the activated and resting states [18], [19]. Therefore, it is likely that different miRNAs and the different sites would specifically modulate each phase of the virus lifecycle in different physiological contexts.

In addition, HIV-1-encoded proteins enhance HIV-1 gene expression over cellular mRNAs by increasing transcription and changing the physiology in the infected cells. Tat can activate transcription through binding the TAR element, present in the R region of the LTR. Vpr can also modulate transcription through the LTR and can induce cell-cycle progression into the G2/M phase, leading to the IRES-oriented expression of mRNAs [58]–[60]. Under these conditions, 5′ 7-methyl guanosine (7 mG) cap-dependent repression by miRNAs is expected to be alleviated [61]. Therefore, when the functions of Tat and Vpr are considered in this context, their ability to promote Rev-mediated relief from silencing seems reasonable (**Fig. S4B**) [58]–[60], [62].

Given that the identified suppressive sites in the *pol* and *env*-*nef* regions appear to be required for the optimal replication or the modulation of HIV-1, we speculate that suppressive sequences might be responsible for the precise subcellular localization of the unspliced viral RNA [17]. Notably, several processing body (P body) components such as APOBEC3G and Mov10 are commonly detected in HIV-1 virions, and the integrity of P bodies appears to be important for HIV-1 virus production [63]–[66]. Furthermore, some positive-strand RNA viruses and retrotransposons use P bodies for genome packaging [67], [68]. Therefore, it is plausible that HIV-1 particles could incorporate several factors if they were located near the P body and the P body was proximal to the packaging site. [63]–[66], [69]. In particular, P bodies recruit aberrant mRNAs and mRNAs that are regulated by RNA interference for destruction, and modulate the expression of mRNAs through storage in discrete loci of the cytoplasm [22], [70], [71]. Therefore, the expression of APOBEC3G might affect the formation of P body structures and lead to the inhibition of miRNA-mediated silencing [72]. Thus, it is also reasonable that silencing of the Rev-mediated mRNA by miRNAs is affected by the overexpression of APOBEC3G, which is not present in HeLa cells (**Fig. S4B** and **C**).

Late endosomal structures called multivesicular bodies (MVBs) and cytoplasmic domains marked by antibodies directed against GW182 (GW bodies) are often juxtaposed with each other and functionally related [15]–[17]. GW bodies have been regarded as P bodies or P body-like structures; however, few studies have adequately distinguished P bodies from GW bodies [15]. Therefore, our results might highlight the importance of the suppressive sequences, which presumably help the viral genome localize to MVBs or P body-like structures [17], [73]. Consequently, the silenced, recruited genome is more likely to coincide with Gag, which is separately recruited to the MVBs [15], [74]–[78]. Thus, a dedicated site for Gag assembly could be created, promoting interactions with ESCRT components and thus encouraging scission at the plasma membrane, which leads to HIV-1 release. In fact, the colocalization of the HIV-1 genome and Gag protein in the MVBs has been observed in T lymphocytes and several epithelial and fibroblast cells [8]–[11]. Late endosomal compartments have also been assumed to serve as productive sites of infectious HIV-1 assembly [8]–[11], [79]. Thus, a more prominent effect would be expected for macrophages, in which viral budding occurs exclusively in the lumen of endosomes. Moreover, several enveloped retroviruses appear to use Rev-like proteins and the late endosome for assembly in the course of budding [80]–[82]. Given that Rev and Rex had similar impacts on the export of unspliced RNAs under miRNA-mediated repression, some cofactors shared by Rev and Rex might be involved in this process [6]. However, considering the observed differences between Rev and Rex and their LTRs and the effects of those differences on their activities and the mediation of miRNA silencing, it seems likely that their functional differences somehow contribute to the level of virus production and disease progression [6], [83].

In addition, Gag has been suggested to stimulate translation from the HIV-1 5′ UTR at low concentrations but to inhibit translation at high concentrations by releasing viral RNA from polysomes and facilitating RNA packaging into nascent virions [84]. Given the observed difference in the amount of Gag production between wild-type and mutant viruses (**Fig. 7E**), the identified suppressive sites might also affect the regulation of Gag expression [85]. However, ***further analysis*** is ***needed*** to clarify these issues. Nonetheless, under the premise that the equilibrium between translation and packaging is important and that cellular cofactors for HIV-1 replication are also miRNA targets [44], the overexpression of a specific miRNA or experiments using knockdowns of RISC complex components to inhibit miRNA synthesis might elucidate different aspects of silencing.

In conclusion, the effects of the repressive sequences that were identified by cloning the genomic regions of the HIV-1 into the 3′ UTR of *Rluc* are modulated by Rev-dependent transport. The effect is limited for unspliced mRNAs. In addition, these previously uncharacterized suppressive sequences function to promote virus production during HIV-1 replication. These results suggest that HIV-1 has evolved to make effective use of silencing during replication, although differences in the amount of virus, the sequence of the binding site and the phase in the viral life cycle might affect other aspects of the innate immune system.

## Materials and Methods

### Plasmid Construction

The psiCHECK-2 vector (Promega) was used to construct reporter vectors to analyze luciferase activity. The pNL4-3 laboratory strain (AF324493) was used to identify silencing loci and to generate mutant viruses. To express Rev-HA and Rev, the pcDNA3.1(+) vector was used. The details of the construction of the plasmids are described in Materials and Methods S1 and Table S2.

### Cell Culture and Transfections

Jurkat, Clone E6-1 cells (ATCC #TIB-152) and Molt-4, Clone 8 (M4C8/MOLT-4^#^8) cells [86] were maintained in RPMI 1640 medium supplemented with 10% fetal bovine serum, 100 U/ml penicillin and 100 µg/ml streptomycin in a humidified 5% CO_2_ atmosphere at 37°C. Targefect-F1 (Targeting Systems) was used for transfection, which was accomplished with a solution containing 0.9 µl of Targefect-F1 and 20–100 ng of plasmid in 150 µl of OPTI-MEM that was vortexed, mixed with 5×10^5^ cells, seeded onto a 48-well plate and incubated for 3 h. The cells were washed with 750 µl of prewarmed media, mixed with 1 ml of prewarmed media and seeded onto a 24-well plate. For cotransfections, 50 ng of the Rev-responsive plasmid and 400 ng of pcDNA3.1(+) vector or vectors expressing Rev-HA or Rev were mixed with 1 µl of Targefect-F1.

HeLa and 293T cells [87], [88] were maintained in Dulbecco’s modified Eagle’s medium supplemented with 10% fetal bovine serum, 100 U/ml penicillin, and 100 µg/ml streptomycin in a humidified, 5% CO_2_ atmosphere at 37°C. For HeLa cell transfections, the cells were trypsinized and seeded onto a 24-well plate at a density of 2×10^4^ cells/well 1 day prior to transfection. Cotransfection was performed using FuGENE6 (Roche Diagnostics) according to the manufacturer’s instructions. For the experiments, 10–20 ng of the psiCHECK-2 vector or the Rev-responsive vectors was cotransfected with 400 ng of the vector expressing either Rev-HA, Rev or a mixture of 200 ng of the Rev-HA/Rev expression plasmid and 200 ng of the vector expressing Tat, Vpr or APOBEC3G. To generate the virus, 293T cells were seeded onto a 24-well plate at a density of 3×10^4^ cells/well 1 day prior to transfection, and 400 ng of pNL4-3 or plasmid expressing each mutant virus was transfected using FuGENE6.

### Luciferase Reporter Assay

After transfection, the cells were incubated for 48 h and lysed in passive lysis buffer (Promega). Firefly and *Renilla* luciferase signals were measured using the Dual Luciferase Reporter Assay System (Promega), and the *Renilla* luciferase activity was normalized to the firefly luciferase activity.

### Nuclear and Cytoplasmic RNA Preparation and Real-time qPCR

HeLa cells were seeded onto a 6-well plate at a density of 1×10^5^ cells/well 1 day prior to transfection. Cotransfection was performed using FuGENE6 (Roche Diagnostics) according to the manufacturer’s instructions. For the experiment, 100 ng of the psiCHECK-2 vector or the Rev-responsive vectors was cotransfected with 2 µg of the vector expressing Rev-HA or pcDNA3.1(+) plasmid. After transfection, the cells were incubated for 48 h, and the nuclear and cytoplasmic RNAs were isolated [89]. Briefly, cells were rinsed with ice-cold phosphate-buffered saline (PBS) and isolated by scraping in 1 ml of ice-cold PBS, and centrifuged at 350×*g* for 5 min at 4°C. The pelleted cells were resuspended in 100 µl of Lysis buffer A [10 mM Tris (pH 8.0), 140 mM NaCl, 1.5 mM MgCl_2_, 0.5% Nonident P-40] and incubated on ice for 5 min. Nuclear pellets were obtained by centrifuging at 1,000×*g* for 3 min at 4°C. The supernatant was transferred to a fresh tube as a cytoplasmic fraction. Nuclear pellets were washed two times with 50 µl of lysis buffer A and finally with lysis buffer A containing 1% Tween-40 and 0.5% deoxycholic acid, and the each supernatant was added to the cytoplasmic fraction. Nuclear pellets were resuspended in 250 µl of lysis buffer A, and Nuclear and cytoplasmic RNAs were isolated and DNase-treated (TURBO DNase, Applied Biosystem) before being finally dissolved in 20 µl of nuclease-free water. cDNAs were synthesized with the ReverTra Ace qPCR RT Kit (TOYOBO). qPCR analysis was performed using specific primer pairs and the *Power* SYBR Green PCR Master Mix (Applied Biosystem). Each sample was analyzed in triplicate. The results were evaluated by the comparative threshold cycle method [90]. The following primers were used: for the *Renilla* luciferase RNA produced from the psiCHECK-2 vector, Rluc-F and Rluc-R; for the intron region in the *Renilla* luciferase RNA, Intron-F and Intron-R; for the firefly luciferase RNA produced from the psiCHECK-2 vector, Fireluc-F and Fireluc-R; for U1snRNA, U1-F and U1-R and for G3PDH, G3PDH-F and G3PDH-R.

### siRNA Transfection

In Jurkat, Clone E6-1 cells, 200 nM of duplex RNAi was transfected into 1×10^6^ cells using an Amaxa nucleofector apparatus and the X-001 program (Amaxa Biosystems). At 48 h post-transfection, 100 ng of the plasmid vector and 100 nM RNAi duplex were cotransfected using the Dharma*FECT* Duo transfection reagent (DHARMACON) according to the manufacturer’s protocol. The following siRNAs were used: anti-Drosha siRNA1 [44], 5′-CGAGUAGGCUUCGUGACUU(dTdT)-3′ (B-Bridge) and anti-Drosha siRNA2, ON-TARGETplus SMARTpool siRNAs, and Human RNASEN (Thermo Scientific). A scrambled version of the Anti-Drosha siRNA1 sequence was used as a control siRNA: 5′-GCGACGUUGGCGUUAUCUA(dTdT)-3′ (B-Bridge).

### Human AGO2 Immunoprecipitations

For the experiment, 2×10^7^ Jurkat, Clone E6-1 cells were collected 48 h after transfection and washed with PBS. The cell pellet was resuspended in lysis buffer [25 mM Tris-HCl (pH 8.0), 150 mM NaCl, 2 mM MgCl_2_, 0.5% NP-40 and 5 mM DTT] with protease inhibitors (Roche Applied Science) and RNase inhibitor (250 U/mL; TOYOBO), lysed on ice for 10 min, and centrifuged at 10,000×*g* at 4°C for 10 min. The aliquots (1/5) of the cleared lysate (input) were kept aside for both RNA extraction and western blot analysis, and the remaining supernatant was saved on ice and preceded to ribonucleoprotein immunoprecipitation [91]. Protein G sepharose beads (GE Healthcare) were rinsed four times with PBS, and mixed with anti-human Ago2, monoclonal antibody (WAKO) or unrelated anti-HA antibody (Roshe) in PBS and rotated for 3 h at 4°C. The antibody-Protein G complexes were blocked with 0.5 mg/mL yeast RNA (Ambion) and 1 mg/mL BSA (Sigma) for further 1 h, and then washed three times in PBS and twice in lysis buffer. The antibody-protein beads were mixed with the cleared cell lysate and rotated for 4 h at 4°C. The beads were washed twice with lysis buffer, three times with lysis buffer containing 900 mM NaCl and 1% NP-40, twice more with lysis buffer and finally washed with lysis buffer containing 0.05% NP-40. The beads were resuspended with lysis buffer and the aliquots (1/10) were used for western blot analysis of the immunoprecipitate (IP). The remaining beads were precipitated and resuspended in Proteinase K buffer [50 mM Tris-Cl (pH 7.4), 150 mM NaCl and 0.05% NP-40]. The suspension was supplemented with Proteinase K and incubated for 30 min at 55°C with agitation. RNAs corresponding to input and IP were extracted, and DNase treatment was performed by using the Turbo DNA-free kit (Ambion) for 30 min at 37°C. cDNAs were synthesized with the ReverTra Ace qPCR RT Kit (TOYOBO). qPCR analysis was performed using specific primer pairs and the *Power* SYBR Green PCR Master Mix (Applied Biosystem). Each sample was analyzed in triplicate. The results were evaluated by the comparative threshold cycle method [90]. The following primers were used: for the *Renilla* luciferase RNA produced from the psiCHECK-2 vector, Rluc-F and Rluc-R and for the firefly luciferase RNA produced from the psiCHECK-2 vector, Fireluc-F and Fireluc-R.

### Virus Infection

Jurkat, Clone E6-1 cells were infected with NL4-3 or mutated virus by mixing 1×10^5^ cells with 100 ng of p24-normalized virus followed by a 1-h incubation at 37°C. The cells were washed in medium three times and then incubated at 37°C. The culture supernatant was collected, and the medium was replaced with the same amount of fresh medium daily. To check for p24 production, a p24 chemiluminescence enzyme immunoassay was used (Lumipulse; Fujirebio, Japan). The cells were infected with each mutated virus and NL4-3 in parallel, and the amount of p24 produced by each mutant virus and NL4-3 was normalized to the level detected in NL4-3-infected cells on day 6. The relative values are presented as the mean ± S.D. For Molt-4, Clone 8 cells, 10 ng of p24-normalized virus was used.

### HIV-1 Indicator Assay

The TZM-bl cell line (#8129) was obtained through the NIH AIDS Research and Reference Reagent Program, Division of AIDS, NIAID, NIH and were contributed by John Kappes and Xiaoyun Wu [46]. The TZM-bl cells were plated in 24-well plates at 4×10^4^ cells/well the day before infection [47]. After removing the medium, 500 µl of fresh medium containing 10 ng of p24-normalized virus was added onto cells in triplicate wells and the cultures were incubated at 37°C. After 40 h, the medium was removed and the cells were washed with PBS and lysed in 100 µl of Passive lysis buffer (Promega). Viral infectivity was quantified by measuring luciferase activity in cell lysates. Experiments were performed four times, and the results were averaged. The results are expressed as the mean ± S.D. as a percentage of NL4-3.

### Western Blot

For western blot analysis, 20 µg of total protein from the transfected cells was separated by SDS–PAGE and transferred onto a Hybond-P PVDF membrane (GE Healthcare). The primary antibodies used were anti-HIV-1 Tat (Santa Cruz Biotechnology, sc-65913; diluted 1/200), anti-Drosha (Abcam, ab12286, diluted 1/400), anti-HA (Santa Cruz Biotechnology, sc-805, diluted 1/100), anti-p24 Gag (NIH AIDS Research and Reference Reagent Program, #24-3, diluted 1/9,000) and anti-β-actin (Sigma, A5441, diluted 1/1,000). The secondary antibodies used were horseradish peroxidase-conjugated anti-mouse IgG (GE Healthcare, NA931V, diluted 1/2,000) and horseradish peroxidase-conjugated anti-rabbit IgG (GE Healthcare, diluted 1/2,000). The bound antibodies were detected using the Enhanced Chemiluminescence Plus Western Blotting Detection System (GE Healthcare).

### MicroRNA Profiling

The total RNA from Jurkat, Clone E6-1 cells cultured under basal conditions was extracted using a mirVana miRNA Isolation Kit (Applied Biosystems). A TaqMan human microRNA assay was performed using a TaqMan Human MicroRNA A&B Array v2.0 (Applied Biosystems Cat#4398977 and Cat#4398978) in an ABI PRISM 7900HT Sequence Detection System, and the data were analyzed using the ABI PRISM SDS 2.1 software (GeneticLab). The RNA-hybrid program (http://bibiserv.techfak.uni-bielefeld.de/rnahybrid/rnahybrid/submission.html) was used to predict the secondary structures of the miRNA/target duplexes.

## Supporting Information

Figure S1 **Validation of the Rev-mediated export of mRNA and the effect of miRNA-mediated silencing.** (A) The expression of Rev-HA in transfected HeLa cells was determined using western blot. As a control, pcDNA3.1(+) was transfected at the same time. The membrane was then stripped and reprobed with anti-ß actin antibody. (B) Validation of the Rev response element (RRE) and Rev-HA function in the presence of a splice donor site (SD). An RRE and an SD were inserted downstream of the stop codon in the *Rluc* gene. The insertion of the inverted RRE is designated as “RRE (−)”. There was no significant difference in the positional relationships of the inserted RRE and SD. (C) The effects of the Bulge and BulgeMut sequences on export using Rev-HA in the context of the presence of the RRE and SD. (D) The effect of the RRE on the RNAs exported by Rev-HA in the absence of the SD. (E) The influence of the Bulge and BulgeMut sequences in the absence of the SD. In each graph, The *Renilla*/firefly luciferase value was assessed, and the data presented are the mean ± S.D. normalized to the empty vector. “pcDNA” denotes the pcDNA3.1(+) plasmid. The red arrow points to the vectors that presented altered *Rluc* activity in the presence of Rev-HA. The red arrowhead points to the Bulge-containing constructs that carry a correctly oriented RRE and were silenced in the presence of Rev-HA.
(TIF)

Figure S2 **The effect of Rev on the cytoplasmic export of RRE-containing RNA.** (A) Analysis of nuclear and cytoplasmic levels of G3PDH RNAs in HeLa cells transfected with each vector (Vectors C, 1, 2, 9 and 10). The indication of the transfected vector corresponds to that in Fig. 1C. The cytoplasmic level of G3PDH RNA was set to 100 in each case. (B) The nuclear and cytoplasmic levels of U1 snRNAs in cells transfected with each vector. The nuclear level of U1 snRNA was set to 100 in each experiment. (C) The nuclear and cytoplasmic levels of firefly luciferase RNAs produced from each transfected vector. The cytoplasmic level of the firefly luciferase RNA was set to 100. (D) The levels of *Rluc* RNAs transported into the cytoplasm were analyzed by RT-qPCR (each normalized to firefly luciferase RNA). The normalized values of *Rluc* RNA levels were expressed as the mean ± S.D. as a percentage of the control psiCHECK vector (C). The red arrow points to the vectors that presented altered *Rluc* activity in the presence of Rev-HA (Fig. 1C) and also presented altered corresponding *Rluc* RNA levels in the cytoplasm in the presence of Rev-HA.
(TIF)

Figure S3 **Validation and confirmation of Rev-mediated RNA export.** (A) The effects of the orientation of the RRE and the presence of the SD were evaluated. (B) The effect of the insertion of the Bulge and BulgeMut sequences on RNA transport by Rev. The *Renilla*/firefly luciferase value was assessed, and the data presented are the mean ± S.D. normalized to the empty vector. “pcDNA” denotes the pcDNA3.1(+) plasmid. The red arrow points to the vectors that presented altered *Rluc* activity in the presence of Rev. The red arrowhead points to the Bulge-containing constructs that carry a correctly oriented RRE and were silenced in the presence of Rev.
(TIF)

Figure S4 **Characterization of possible effects on** **silencing activity during Rev-dependent export.** (A) The effect of the 3×Bulge and Perfect sequences on the silencing of Rev-HA-exported RNAs. The inverted RRE insertion is designated as “RRE (−)”. The blue and dashed blue arrows and blue arrowhead point to the Bulge-, 3×Bulge- or Perfect-containing constructs in the presence of Rev-HA individually. (B) Potential modulators that inhibit miRNA-mediated silencing during Rev-HA export. In addition to Rev-HA, the plasmids expressing APOBEC3G (Apo), Tat or Vpr were individually cotransfected into HeLa cells, and these cells were compared with cells that were transfected with Rev-HA alone (dashed blue arrows). (C) The effects of Rev-HA, Apo, Tat and Vpr on RNAs containing sequences targeted by let-7 but not RRE or SD sequences.
(TIF)

Figure S5 **Schematic representation of the suppressive regions present in the HIV-1 genome.** (A) HIV-1 DNA fragments amplified from pNL4-3 (AF324493) were inserted into the *Rluc* 3′ UTR present in the psiCHECK. The plasmid was transfected into Jurkat cells, and the cell lysates were prepared at 48 h post-transfection. Luciferase activity was assessed using the Dual Luciferase Reporter Assay System, and the *Rluc* activity was normalized to the firefly luciferase activity. An empty psiCHECK was used as a control. The figure presents the data averaged from three independent transfections. The degree of repression is represented as a patterned bar below the indicated genomic region. (B) M4C8 cells were transfected and analyzed as described in (A).
(TIF)

Figure S6 **The identification of suppressive sequences in the** ***pol*** **and** ***env*** **-** ***nef*** **regions.** (A) The regions in *pol* and *env*-*nef* that were identified as susceptible to silencing are illustrated. The number and bar pattern corresponds to the graph below. (B) The suppressive regions in the *pol* and *env*-*nef* gene were assessed by dissection of the region and analysis in Jurkat cells. The *Renilla*/firefly luciferase value was assessed, and the data shown are the mean percentages ± S.D. of the activity in the empty psiCHECK.
(TIF)

Figure S7 **The characterization of the suppressive sequence in the** ***env*** **-** ***nef*** **region.** The underlined sequences in sites “d”, “28″, and the corresponding portion of “28-m” were concatenated and assessed in Jurkat cells. The gray characters indicate the unchanged residues. The *Rluc* activity was normalized to the firefly luciferase activity. Three independent experiments were performed and the data shown are as the mean percentages ± S.D. of the activity in the empty psiCHECK. *P<0.05.
(TIF)

Figure S8 **The effects of combining multiple mutations in other cells.** (A) The combination of the mutations in the *pol* and *env*-*nef* regions that demonstrated relief from silencing in Jurkat and M4C8 cells was also validated in HeLa cells. (B) The effects of combined *pol* and *env*-*nef* region mutations in 293T cells. In each graph, the *Rluc* activity was normalized to the firefly luciferase activity. The psiCHECK was used as a control, and the results are the average data from six independent transfections and expressed as the mean ± S.D. as a percentage of the control. ***P<0.001. The bar patterns in the graph are the same as those in Fig. 5.
(TIF)

Figure S9 **The effect of the HIV-1 and HTLV-1 promoters on Rev and Rex function.** (A) Schematics of the pU3, pU3IN and pU3 (HTLV) constructs. (B) The function of Rex was analyzed using the U3 regions of HIV-1 and HTLV-1, and the effect of splicing was also evaluated by comparing the level of silencing relief by pU3 and pU3IN. The blue arrow points to the Bulge-containing constructs without an intron that carry a correctly oriented RRE and were not silenced in the presence of Rex alone. The red arrowhead points to the Bulge-containing constructs that carry an intron and a correctly oriented RRE and were silenced in the presence of Rex. The blue arrowhead points to the construct without an intron that was silenced in the presence of Rex alone. The U3 regions of HTLV-1 and HIV-1 were also evaluated in the presence or absence of Tat or Tax. Three independent experiments were performed. **P<0.005. (C) The comparison of the Rev-HA and Rex activities on the BulgeMut-containing constructs. The red arrow points to the vectors that presented altered *Rluc* activity in the presence of Rev-HA or Rex. (D) The effect of Tax on the U3 (HTLV) promoter. Rev-HA function was also assessed in this promoter context (blue arrows). “C” denotes the empty vector used as a control in each promoter context. The *Renilla*/firefly luciferase values are expressed as the mean ± S.D. as a percentage of the control.
(TIF)

Table S1 **Sequences and amino acids for each mutated pattern.** (A) Patterns in the *pol* region. The changed sequences and amino acids correspond to sites “a” and “b” in Fig. 4A, E and Fig. 5A, F. (B) Patterns in the *env*-*nef* region. The changed sequences and amino acids correspond to sites “c”, “d” and “e” in Fig. 4A, E and Fig. 5A, G. The gray characters indicate unchanged residues, their encoded amino acids and their frequency per thousand codons. Asterisks denote mutations that did not change any amino acids.
(TIF)

Table S2 **Primers and oligonucleotides.** (A) Primer sequences. (B) Oligonucleotide sequences.
(TIF)

Materials and Methods S1 **Plasmid construction.**
(PDF)
