# Supplementary figures and images for: HIV-1 Suppressive Sequences Are Modulated by Rev Transport of Unspliced RNA and Are Required for Efficient HIV-1 Production
Source: PLoS One. 2012 Dec 10;7(12):e51393. doi: 10.1371/journal.pone.0051393 (PMC3519575; doi:10.1371/journal.pone.0051393)

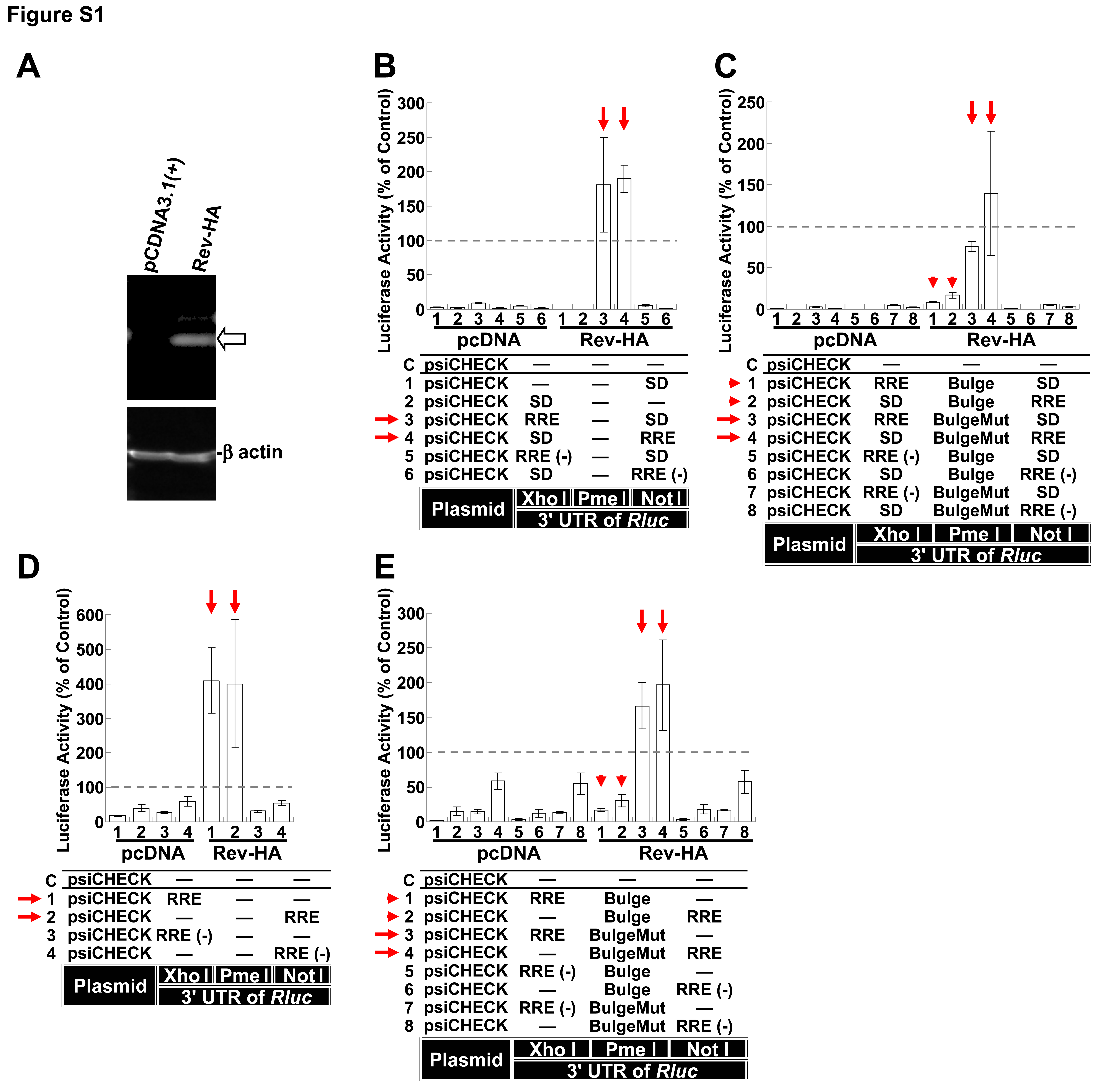

Supplement: Figure S1 — Validation of the Rev-mediated export of mRNA and the effect of miRNA-mediated silencing. (A) The expression of Rev-HA in transfected HeLa cells was determined using western blot. As a control, pcDNA3.1(+) was transfected at the same time. The membrane was then stripped and reprobed with anti-ß actin antibody. (B) Validation of the Rev response element (RRE) and Rev-HA function in the presence of a splice donor site (SD). An RRE and an SD were inserted downstream of the stop codon in the Rluc gene. The insertion of the inverted RRE is designated as “RRE (−)”. There was no significant difference in the positional relationships of the inserted RRE and SD. (C) The effects of the Bulge and BulgeMut sequences on export using Rev-HA in the context of the presence of the RRE and SD. (D) The effect of the RRE on the RNAs exported by Rev-HA in the absence of the SD. (E) The influence of the Bulge and BulgeMut sequences in the absence of the SD. In each graph, The Renilla/firefly luciferase value was assessed, and the data presented are the mean ± S.D. normalized to the empty vector. “pcDNA” denotes the pcDNA3.1(+) plasmid. The red arrow points to the vectors that presented altered Rluc activity in the presence of Rev-HA. The red arrowhead points to the Bulge-containing constructs that carry a correctly oriented RRE and were silenced in the presence of Rev-HA. (TIF) [file pone.0051393.s001.tif]

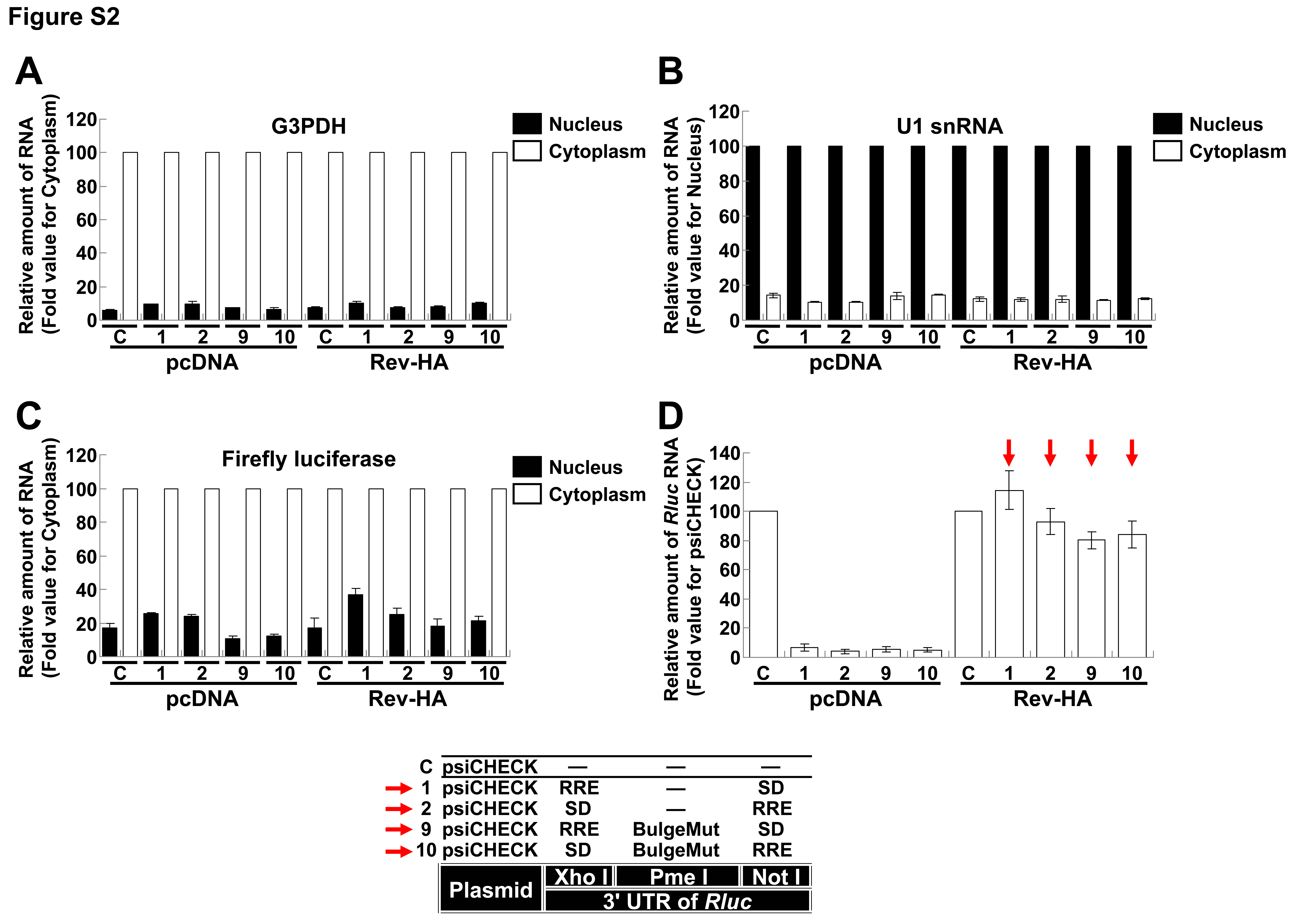

Supplement: Figure S2 — The effect of Rev on the cytoplasmic export of RRE-containing RNA. (A) Analysis of nuclear and cytoplasmic levels of G3PDH RNAs in HeLa cells transfected with each vector (Vectors C, 1, 2, 9 and 10). The indication of the transfected vector corresponds to that in Fig. 1C. The cytoplasmic level of G3PDH RNA was set to 100 in each case. (B) The nuclear and cytoplasmic levels of U1 snRNAs in cells transfected with each vector. The nuclear level of U1 snRNA was set to 100 in each experiment. (C) The nuclear and cytoplasmic levels of firefly luciferase RNAs produced from each transfected vector. The cytoplasmic level of the firefly luciferase RNA was set to 100. (D) The levels of Rluc RNAs transported into the cytoplasm were analyzed by RT-qPCR (each normalized to firefly luciferase RNA). The normalized values of Rluc RNA levels were expressed as the mean ± S.D. as a percentage of the control psiCHECK vector (C). The red arrow points to the vectors that presented altered Rluc activity in the presence of Rev-HA (Fig. 1C) and also presented altered corresponding Rluc RNA levels in the cytoplasm in the presence of Rev-HA. (TIF) [file pone.0051393.s002.tif]

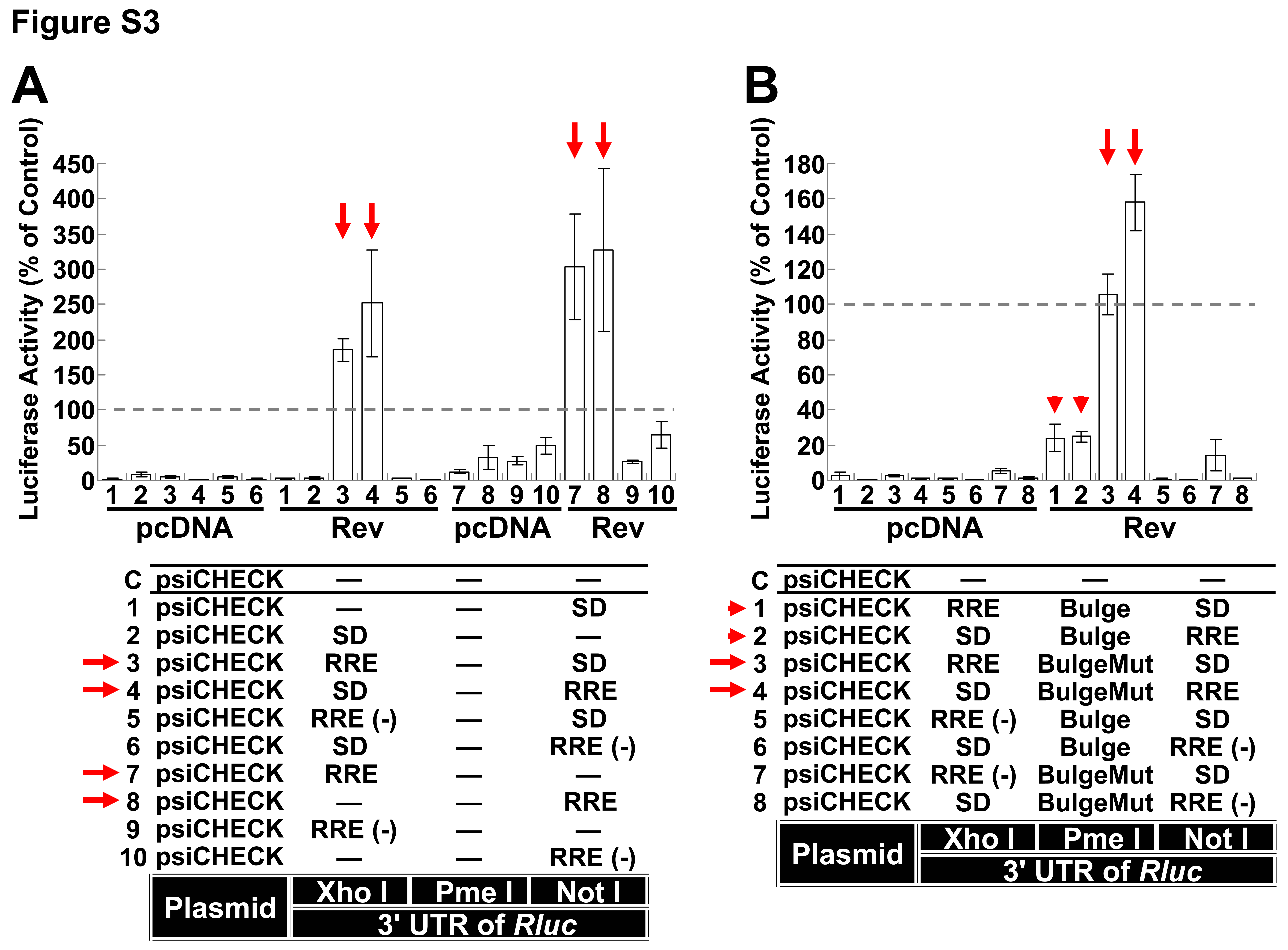

Supplement: Figure S3 — Validation and confirmation of Rev-mediated RNA export. (A) The effects of the orientation of the RRE and the presence of the SD were evaluated. (B) The effect of the insertion of the Bulge and BulgeMut sequences on RNA transport by Rev. The Renilla/firefly luciferase value was assessed, and the data presented are the mean ± S.D. normalized to the empty vector. “pcDNA” denotes the pcDNA3.1(+) plasmid. The red arrow points to the vectors that presented altered Rluc activity in the presence of Rev. The red arrowhead points to the Bulge-containing constructs that carry a correctly oriented RRE and were silenced in the presence of Rev. (TIF) [file pone.0051393.s003.tif]

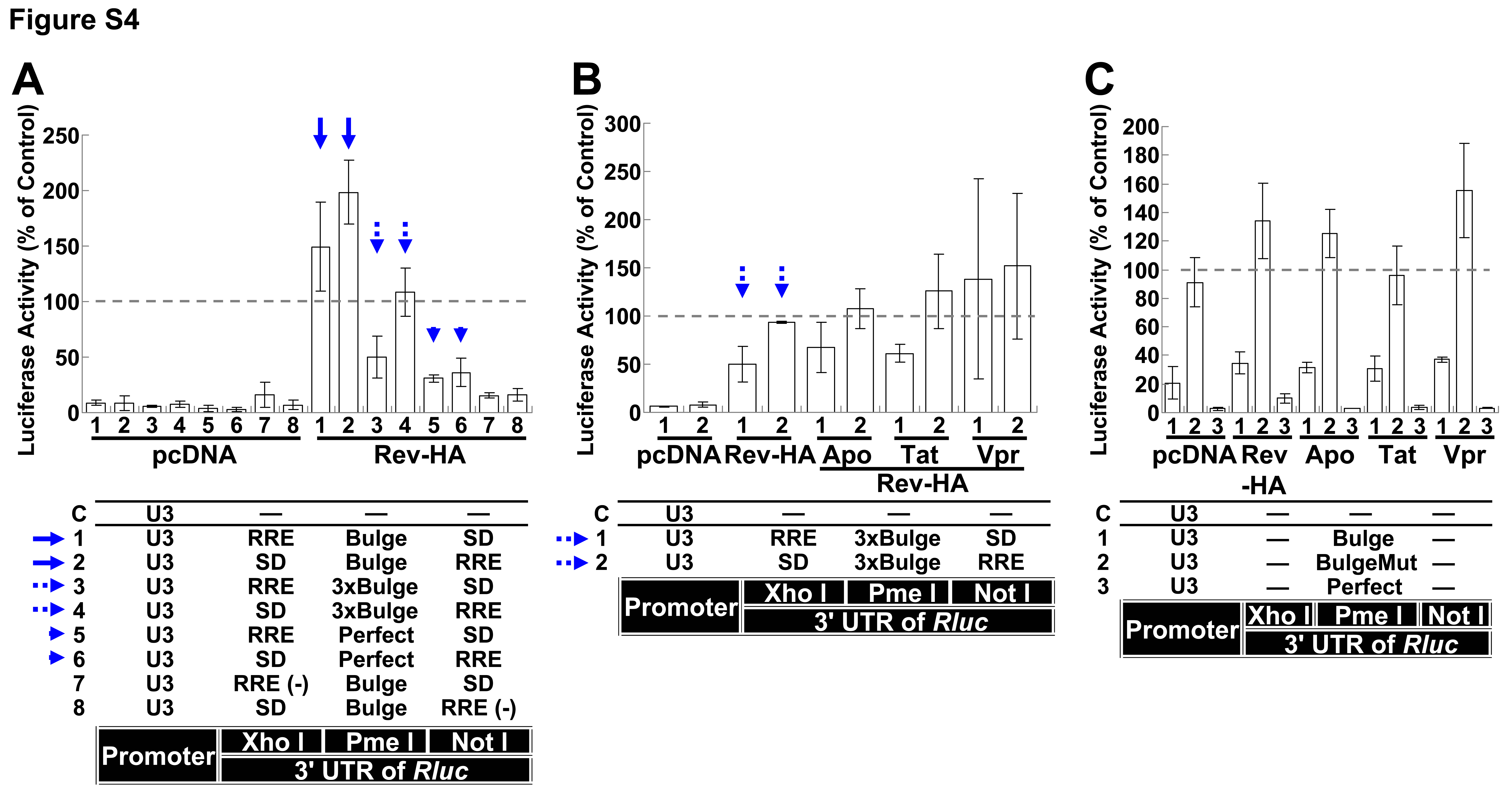

Supplement: Figure S4 — Characterization of possible effects on silencing activity during Rev-dependent export. (A) The effect of the 3×Bulge and Perfect sequences on the silencing of Rev-HA-exported RNAs. The inverted RRE insertion is designated as “RRE (−)”. The blue and dashed blue arrows and blue arrowhead point to the Bulge-, 3×Bulge- or Perfect-containing constructs in the presence of Rev-HA individually. (B) Potential modulators that inhibit miRNA-mediated silencing during Rev-HA export. In addition to Rev-HA, the plasmids expressing APOBEC3G (Apo), Tat or Vpr were individually cotransfected into HeLa cells, and these cells were compared with cells that were transfected with Rev-HA alone (dashed blue arrows). (C) The effects of Rev-HA, Apo, Tat and Vpr on RNAs containing sequences targeted by let-7 but not RRE or SD sequences. (TIF) [file pone.0051393.s004.tif]

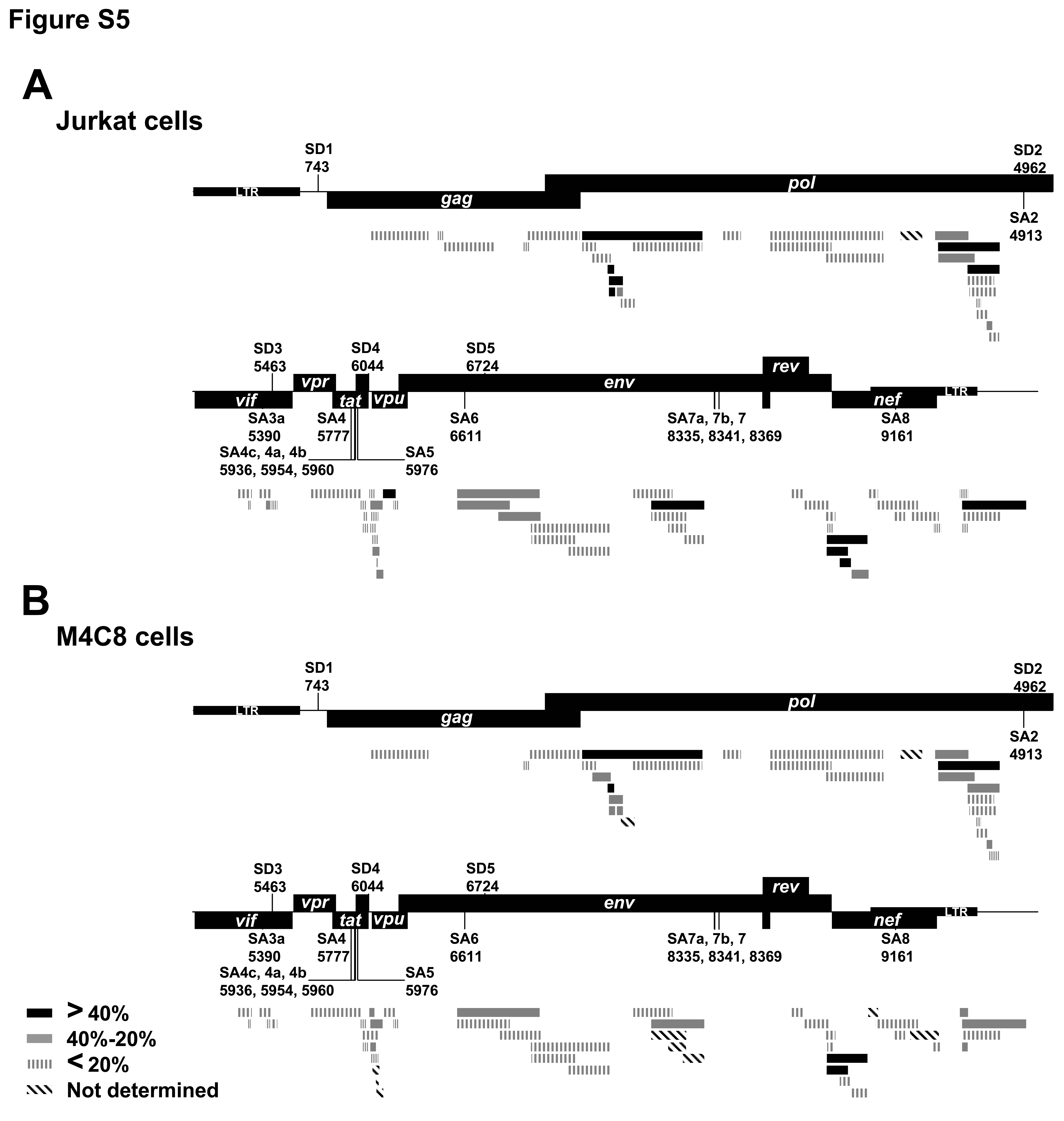

Supplement: Figure S5 — Schematic representation of the suppressive regions present in the HIV-1 genome. (A) HIV-1 DNA fragments amplified from pNL4-3 (AF324493) were inserted into the Rluc 3′ UTR present in the psiCHECK. The plasmid was transfected into Jurkat cells, and the cell lysates were prepared at 48 h post-transfection. Luciferase activity was assessed using the Dual Luciferase Reporter Assay System, and the Rluc activity was normalized to the firefly luciferase activity. An empty psiCHECK was used as a control. The figure presents the data averaged from three independent transfections. The degree of repression is represented as a patterned bar below the indicated genomic region. (B) M4C8 cells were transfected and analyzed as described in (A). (TIF) [file pone.0051393.s005.tif]

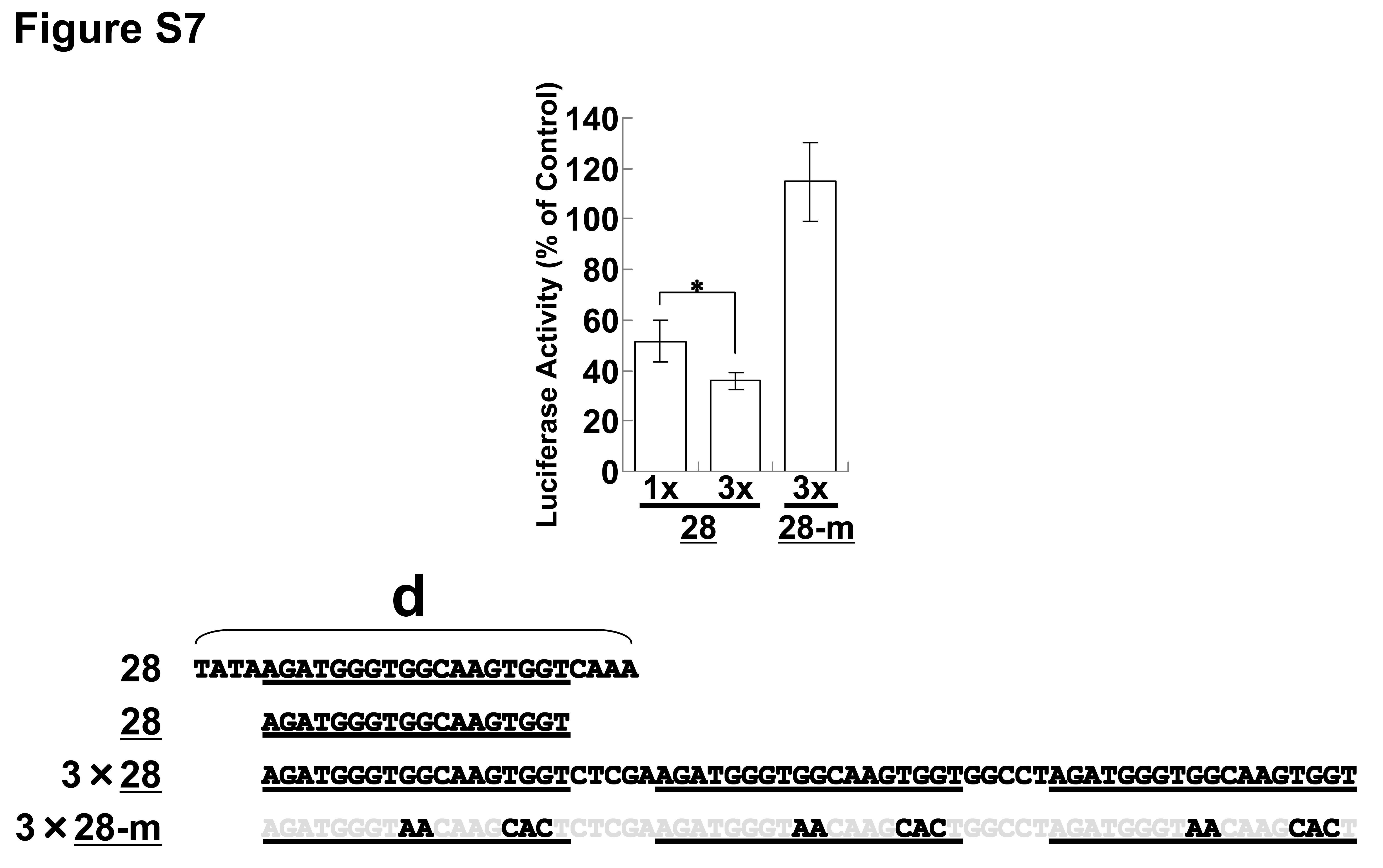

Supplement: Figure S7 — The characterization of the suppressive sequence in the env - nef region. The underlined sequences in sites “d”, “28″, and the corresponding portion of “28-m” were concatenated and assessed in Jurkat cells. The gray characters indicate the unchanged residues. The Rluc activity was normalized to the firefly luciferase activity. Three independent experiments were performed and the data shown are as the mean percentages ± S.D. of the activity in the empty psiCHECK. *P<0.05. (TIF) [file pone.0051393.s007.tif]

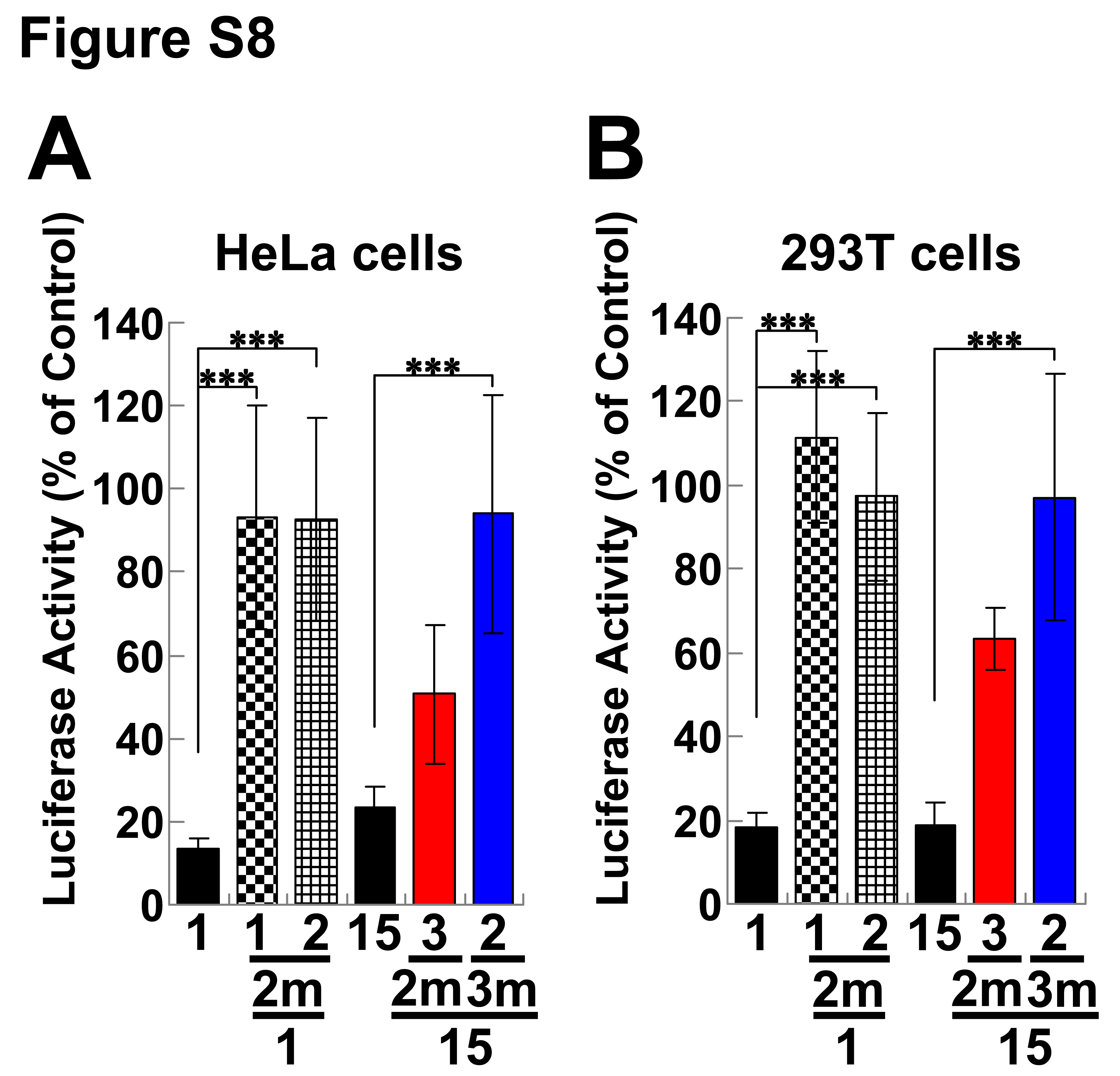

Supplement: Figure S8 — The effects of combining multiple mutations in other cells. (A) The combination of the mutations in the pol and env-nef regions that demonstrated relief from silencing in Jurkat and M4C8 cells was also validated in HeLa cells. (B) The effects of combined pol and env-nef region mutations in 293T cells. In each graph, the Rluc activity was normalized to the firefly luciferase activity. The psiCHECK was used as a control, and the results are the average data from six independent transfections and expressed as the mean ± S.D. as a percentage of the control. ***P<0.001. The bar patterns in the graph are the same as those in Fig. 5. (TIF) [file pone.0051393.s008.tif]

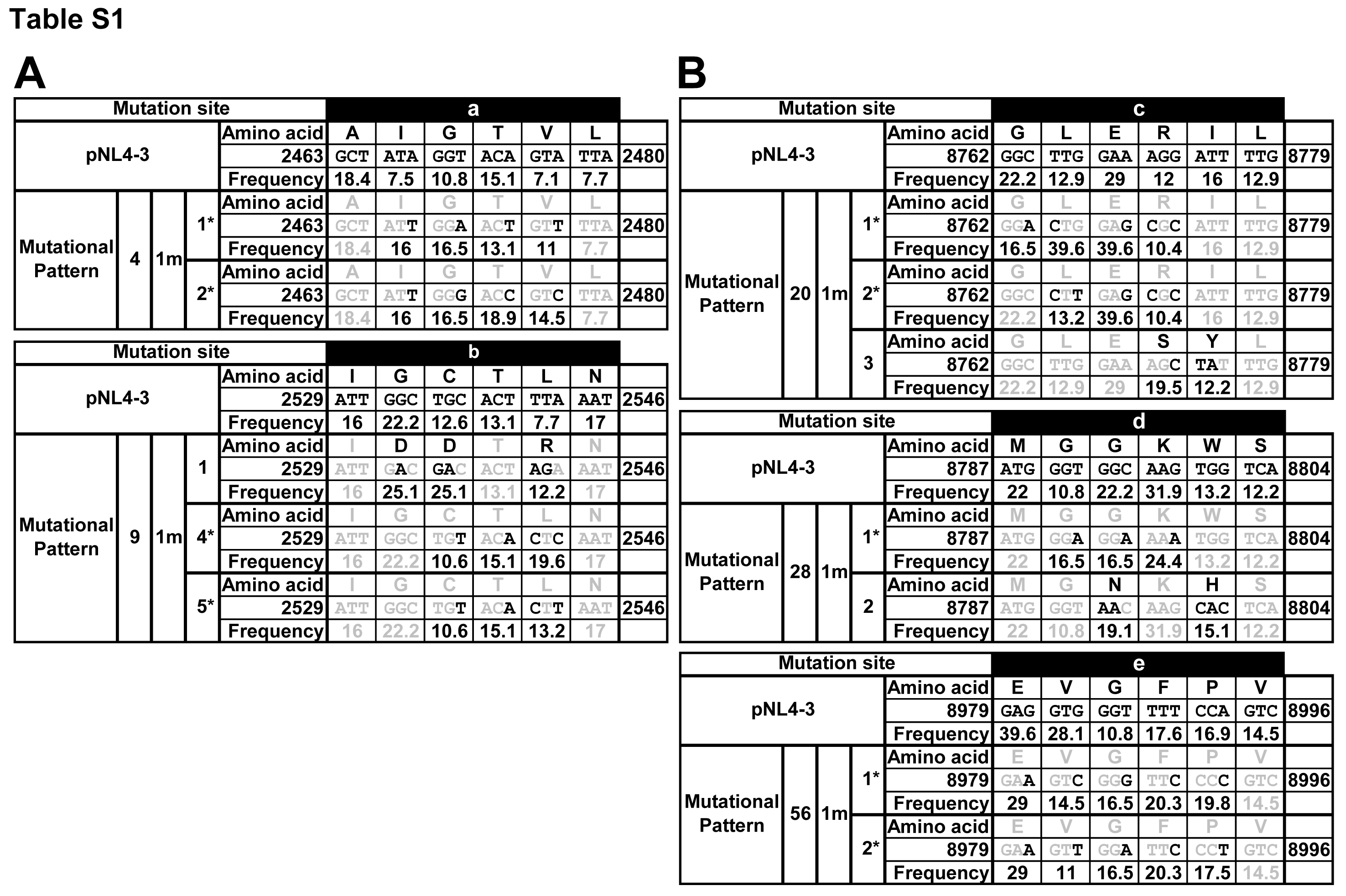

Supplement: Table S1 — Sequences and amino acids for each mutated pattern. (A) Patterns in the pol region. The changed sequences and amino acids correspond to sites “a” and “b” in Fig. 4A, E and Fig. 5A, F. (B) Patterns in the env-nef region. The changed sequences and amino acids correspond to sites “c”, “d” and “e” in Fig. 4A, E and Fig. 5A, G. The gray characters indicate unchanged residues, their encoded amino acids and their frequency per thousand codons. Asterisks denote mutations that did not change any amino acids. (TIF) [file pone.0051393.s010.tif]

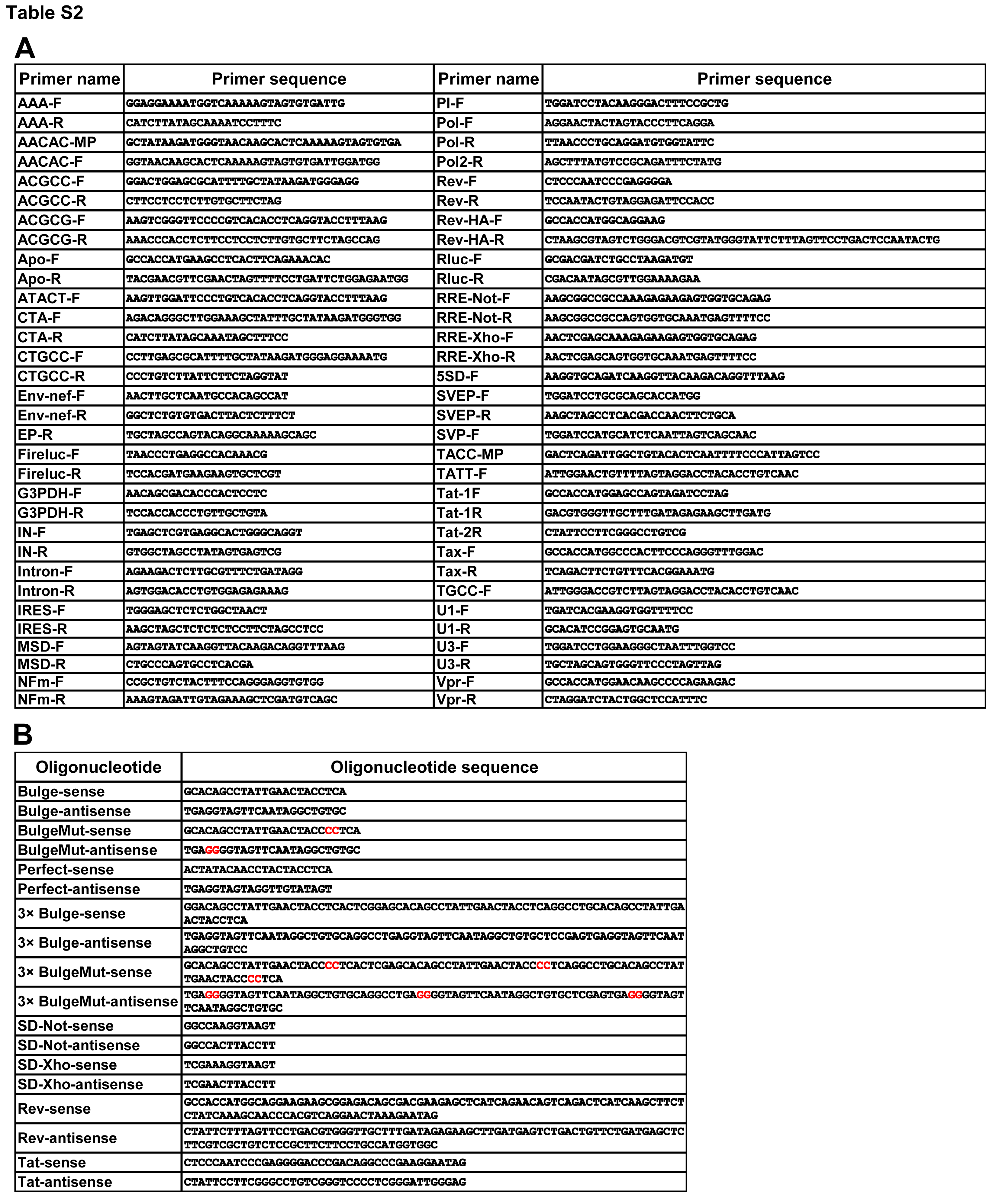

Supplement: Table S2 — Primers and oligonucleotides. (A) Primer sequences. (B) Oligonucleotide sequences. (TIF) [file pone.0051393.s011.tif]
