# Supplementary material for: HIV-1 Suppressive Sequences Are Modulated by Rev Transport of Unspliced RNA and Are Required for Efficient HIV-1 Production
Source: PLoS One. 2012 Dec 10;7(12):e51393. doi: 10.1371/journal.pone.0051393 (PMC3519575; doi:10.1371/journal.pone.0051393)
Supplement: Materials and Methods S1 — Plasmid construction. (PDF) [file pone.0051393.s012.pdf]

Materials and Methods S1

Plasmid construction

The primers and oligonucleotides used to construct the vectors are listed in supporting Table S2A and B. For the identification of suppressive regions in the HIV-1 genome, annealed oligonucleotides or DNA fragments amplified using PCR with pNL4-3 (AF324493) as a template were inserted into the PmeI site of the psiCHECK-2 vector (Promega). For the construction of the plasmids mutated in the *pol* region, annealed oligonucleotides (for “4-1m-1, -2, -3” and “9-1m-1, -2, -3, -4, -5”) and fragments amplified using mutated primers (for “7-2m-1, -2”) were inserted into the PmeI site of the psiCHECK-2 vector. For “1-2m-1, 2”, a mutated pNL4-3 was used as the template for PCR.

To generate viruses with mutations in the *pol* region, the region was PCR amplified using Pol-F and Pol-R primers and pNL4-3 as the template; the resulting fragment was inserted into the EcoRV site of pVAX1. The resulting plasmid was subsequently used to introduce specific mutations using the QuikChange Multi Site-Directed Mutagenesis Kit (Stratagene) and the KOD Plus Mutagenesis Kit (TOYOBO) according to the manufacturers’ instructions. The mutated plasmids used to produce mutated virus were PCR amplified using the same primer sets as those used for insertion into pVAX1. The amplified DNA fragments were digested with SpeI and SbfI and ligated into the SpeI and SbfI sites of digested pNL4-3. For the “9-1m-4” mutation, TACC-MP was used as the primer. For the “4-1m-1” mutation, TATT-F and Pol2-R were used as the primers. For the “4-1m-2” mutation, TGCC-F and Pol2-R were used as the primers.

Of the plasmids mutated in the *env-nef* region, “20-1m-1, -2, -3”, “28-1m-1, -2”, “29-1m-1, -2”, “46-1m-1” and “56-1m-1, -2, -3, -4” were constructed using annealed oligonucleotides containing the mutations. For “16-1m-1, -2, -3, -4, -5, -6”, “16-2m-1, -2, -3”, “16-3m-1, -2, -3, -4, -5”, “15-2m-2, -3” and “15-3m-1, -2, -3, -4, -5”, pVAX1 plasmids mutated in the *env-nef* region were used as templates for PCR, and mutated primers were used for the amplification. Site-directed mutagenesis of the *env-nef* region was conducted as described for the *pol* region. The DNA fragment covering the *env-nef* region was initially PCR amplified using Env-nef-F and Env-nef-R as the primers and then inserted into the EcoRV site of pVAX1. This plasmid was then used as the template for site-directed mutagenesis. For the “20-1m-3” and “16-1m-3” mutations, the CTA-F primer was used. For the “28-1m-2” and “16-1m-5” mutations, the AACAC-MP primer was used. For the “16-3m-5” and “15-3m-5” mutations, the AACAC-F and CTA-R primers were used. For the “46-1m-1” and “56-1m-1” mutations, the ACGCG-F and ACGCG-R primers were used. For the “28-1m-1” and “16-1m-4” mutations, the AAA-F and AAA-R primers were used. For the “56-1m-2” and “16-1m-6” mutations, the ATACT-F and ACGCC-R primers were used. For the “16-3m-3” mutation, the CTGCC-F and CTGCC-R primers were used. For the “20-1m-1” and “16-1m-1” mutations, the ACGCC-F and ACGCC-R primers were used.

To generate mutated viruses, the pVAX1 plasmids carrying mutations in the *env-nef* region were used as a template for PCR amplification using the same primer sets that were used for insertion into pVAX1. The resulting fragments were digested with NgoMIV and inserted into pNL4-3 digested with HpaI and NgoMIV.

For the construction of Rev-responsive vectors, annealed sense and antisense oligonucleotides were inserted into the 3’ UTR of *Renilla* luciferase in the psiCHECK-2 vector. The let-7 targeting sequences, which were reported previously, were inserted into

the PmeI site [22]. The BulgeMut sequence has a 2-nt insertion (shown in red in supporting Table S2B) in the seed region of the bulge let-7 complementary site. The splice donor site (SD) was inserted into the NotI or XhoI sites. For the NotI site, SD-Not-sense and SD-Not-antisense oligonucleotides were used. For the XhoI site, SD-Xho-sense and SD-Xho-antisense oligonucleotides were used. The DNA fragment (7,716-8,034) corresponding to the Rev response element (RRE) of pNL4-3 was PCR amplified and digested with NotI or XhoI and inserted into the NotI site or XhoI site of the *Renilla* luciferase 3' UTR. For the NotI site, RRE-Not-F and RRE-Not-R were used as the primers. For the XhoI site, RRE-Xho-F and RRE-Xho-R were used as primers.

For the plasmid expressing Rev, annealed Rev sense and antisense oligonucleotides were inserted into the PmeI site of pcDNA3.1(+). The resulting vector was digested with BmgBI and ligated with a DNA fragment that was PCR amplified using Rev-F and Rev-R primers and pNL4-3 as the template. For the plasmid expressing Rev-HA, the Rev-expressing fragment was PCR amplified using Rev-HA-F and Rev-HA-R primers and inserted into the PmeI site of pcDNA3.1(+). To construct the Tat-expressing vector, a DNA fragment was PCR amplified using Tat-1F and Tat-1R primers and pNL4-3 as the template. The amplified fragment was inserted into the EcoRV site of pVAX1. The resulting vector was subsequently digested with BmgBI and ligated with the annealed Tat-sense and antisense oligonucleotides. The resulting vector was then PCR amplified using Tat-1F and Tat-2R primers, and the resulting fragment was inserted into the PmeI site of pcDNA3.1(+). For the Vpr-expressing vector, the coding region was PCR amplified using Vpr-F and Vpr-R primers and pNL4-3 as the template, and the resulting fragment was subsequently inserted into the PmeI site of the pcDNA3.1(+) plasmid. Human APOBEC3G was PCR amplified using Apo-F and Apo-R primers, and the resulting fragment was inserted into the PmeI site of pcDNA3.1(+). pSRαΔRex was constructed from the pSRαRex plasmid, which produce the Rex of HTLV-1 but not produce Tax, kindly provided by Dr. Hisatoshi Shida. The original plasmid was digested with PstI-EcoRI, the ends were blunted and the plasmid was self-ligated. The Tax expression plasmid was constructed by PCR-amplifying pCMVHT-ΔEnv using the Tax-F and Tax-R primers and inserting the resulting fragment into the PmeI site of pcDNA3.1(+).

To construct the "pSVEP" and "pSVP" vectors, the psiCHECK-2 vector was amplified using SVEP-F and SVEP-R primers for pSVEP or SVP-F and SVEP-R primers for pSVP. The PCR-amplified fragments were digested with BamHI and NheI and inserted into the BglII-NheI site of the psiCHECK-2 vector. The constructs carrying the inserted RRE and SD in the *Renilla* luciferase 3' UTR were selected. For the "pSVEPI" vector, a fragment amplified from the internal ribosome entry site (IRES) of the LTR using the IRES-F and IRES-R primers was digested with SacI and NheI and inserted into the SacI-NheI site of the "pSVEP" vector. Constructs carrying the inserted sequence such as Bulge, BulgeMut, RRE and SD were selected.

To construct the vectors containing the LTR as the promoter, the pNL4-3-del construct, which carries a deletion in the 3' LTR portion of pNL4-3, was used as the template for PCR amplification. For the "U3" construct, U3-F and U3-R were used as primers. For the "EP" construct, the U3-F and EP-R primers were used. For the "PI" construct, PI-F and IRES-R were used as primers. For the "PT" construct, the PI-F and U3-R primers were used. For the "P" construct, the PI-F and EP-R primers were used. The PCR-amplified fragments were digested with BamHI and NheI and inserted into the BglII-NheI site of psiCHECK-2.

The constructs carrying the sequence such as Bulge, BulgeMut, RRE and SD inserted in the *Renilla* luciferase 3' UTR were then selected. For the "U3I" construct, a DNA fragment was PCR amplified from the IRES region of the LTR, digested with SacI and NheI and inserted into the SacI-NheI site of the "U3" construct. Constructs carrying the inserted sequence such as Bulge, BulgeMut, RRE and SD were then selected. To abrogate the function of the NF-κB sites, the KOD-Plus-Mutagenesis Kit was used with NFm-F and NFm-R primers. To construct the "U3IN" construct, a DNA fragment that was PCR amplified from the intron of psiCHECK-2 using IN-F and IN-R as primers was digested with SacI and NheI and inserted into the SacI-NheI site of the "U3" construct. Constructs carrying the inserted sequence such as Bulge, BulgeMut, RRE and SD were then selected. To abrogate the function of the SD of psiCHECK-2, the KOD-Plus-Mutagenesis Kit was used. To construst the pmSD vector, MSD-F and MSD-R primers were used. To construct the p5SD vector, 5SD-F and MSD-R primers were used. The resulting constructs were digested with StuI and NheI. The extracted DNA fragments were inserted into the StuI-NheI site of the psiCHECK-2 vectors carrying the inserted sequence such as Bulge, BulgeMut, RRE and SD. All subcloned and mutated sequences were verified by DNA sequencing.
